# Supplementary material for: The genetic architecture of host response reveals the importance of arbuscular mycorrhizae to maize cultivation
Source: eLife. 2020 Nov 19;9:e61701. doi: 10.7554/eLife.61701 (PMC7676867; doi:10.7554/eLife.61701)
Supplement: Figure 3—source data 1. — LOD plots of the single-scan QTL performed on the 17 traits studied (see Supplementary file 1 Table S2 for trait codes). The color of the line represents the different models considered: AMF-S families only (H0wt, blue line), AMF-R only (H0hun, red line), AMF as an additive covariate (Ha, yellow line), AMF as an interactive covariate (Hf, purple line), and the evidence of interaction (Hi, green line). The solid line represents the QTL and drop 1-LOD confidence interval. The horizontal dotted line represents the LOD threshold obtained with a 1000 permutations(α = 0.05) for H0wt, H0hun, Ha, and Hf and calculated as LOD_thri = LOD_thrf - LOD_thra for Hi. [file elife-61701-fig3-data1.pdf]

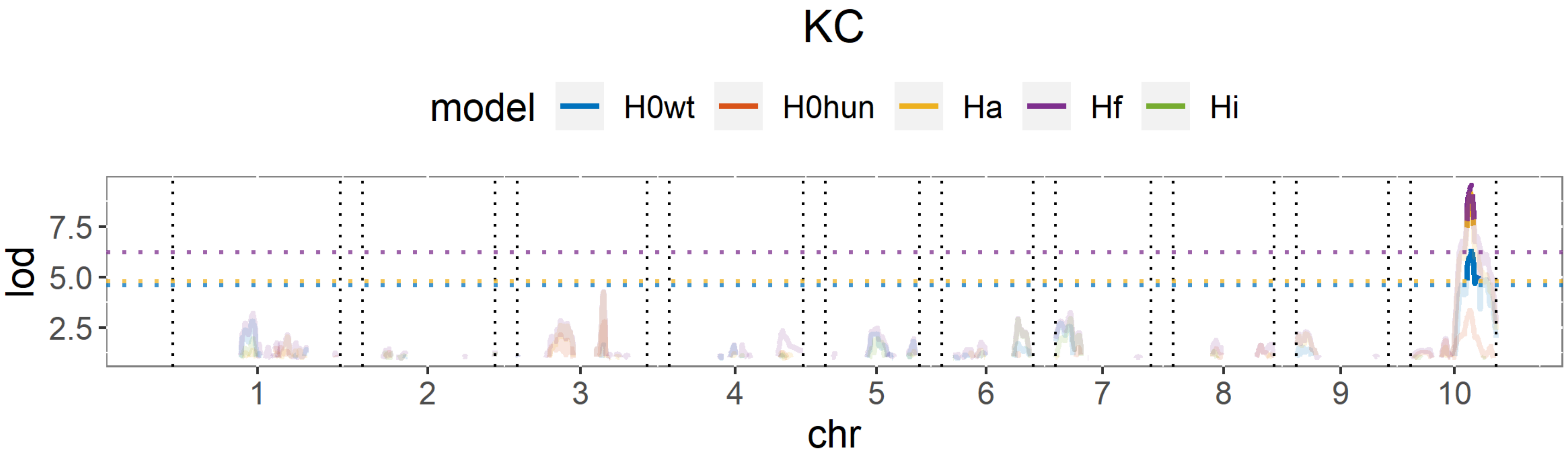

KC
model
H0wt
H0hun
Ha
Hf
Hi
lod
7.5
5.0
2.5
1
2
3
4
5
6
7
8
9
10
chr

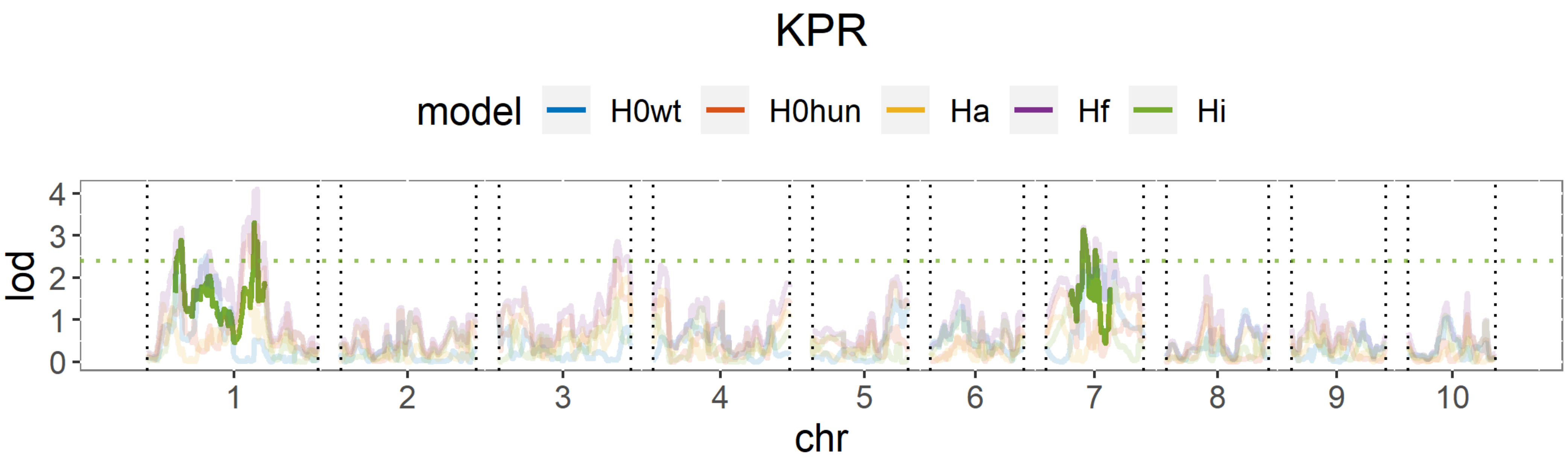
KPR
model
H0wt
H0hun
Ha
Hf
Hi
lod
0
1
2
3
4
1
2
3
4
5
6
7
8
9
10
chr

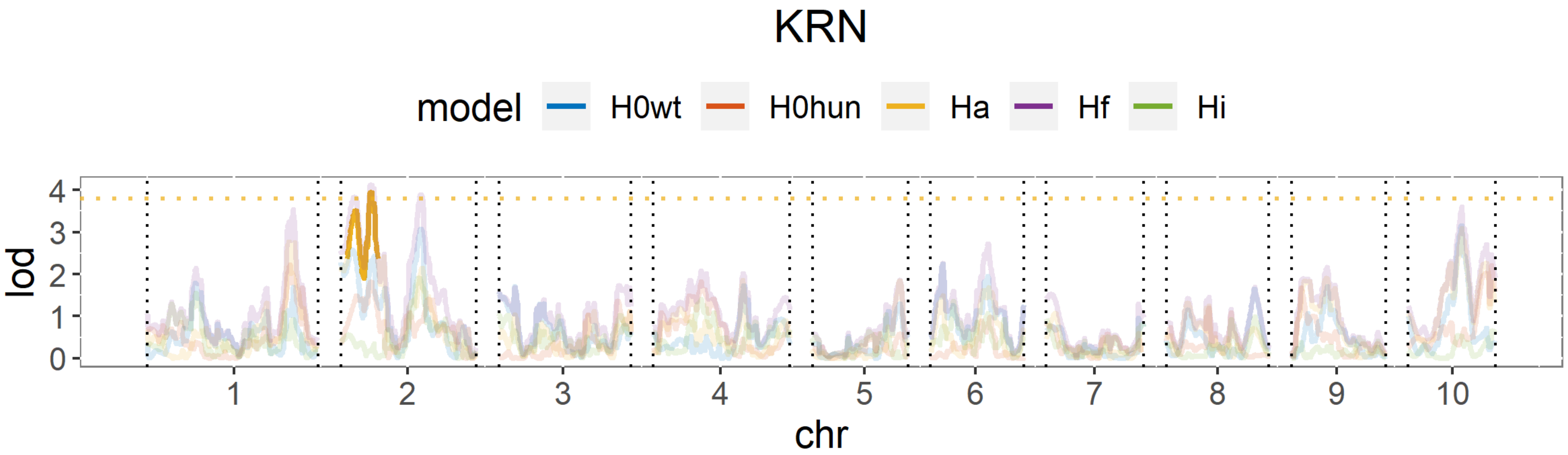

KRN
model
H0wt
H0hun
Ha
Hf
Hi
lod
0
1
2
3
4
chr
1
2
3
4
5
6
7
8
9
10

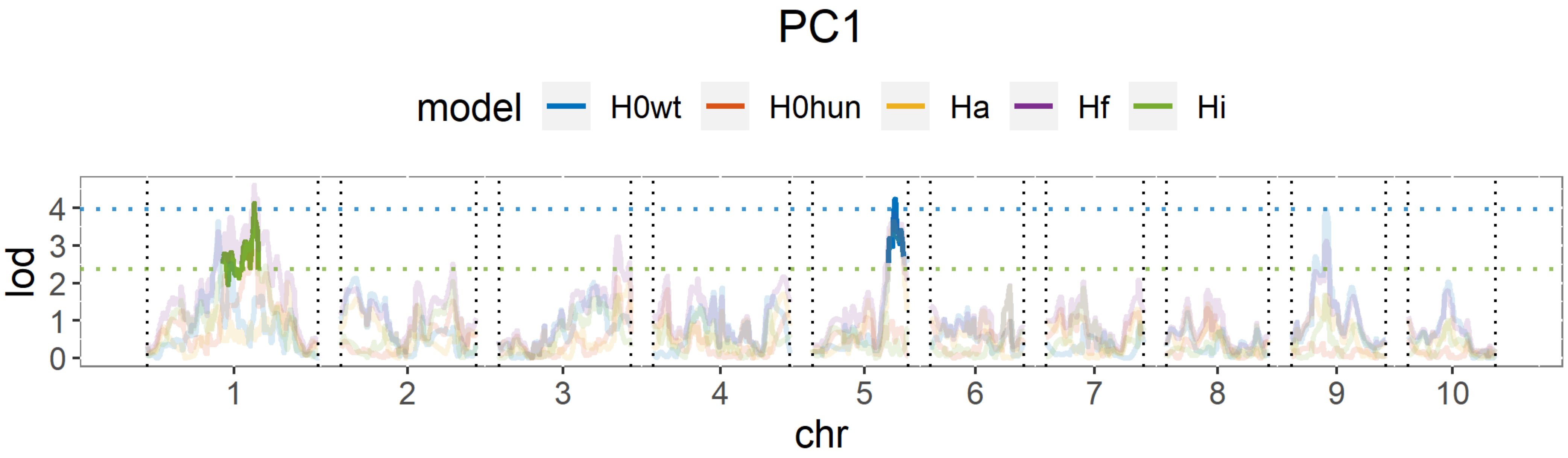

PC1
model
H0wt
H0hun
Ha
Hf
Hi
lod
4
3
2
1
0
1
2
3
4
5
6
7
8
9
10
chr

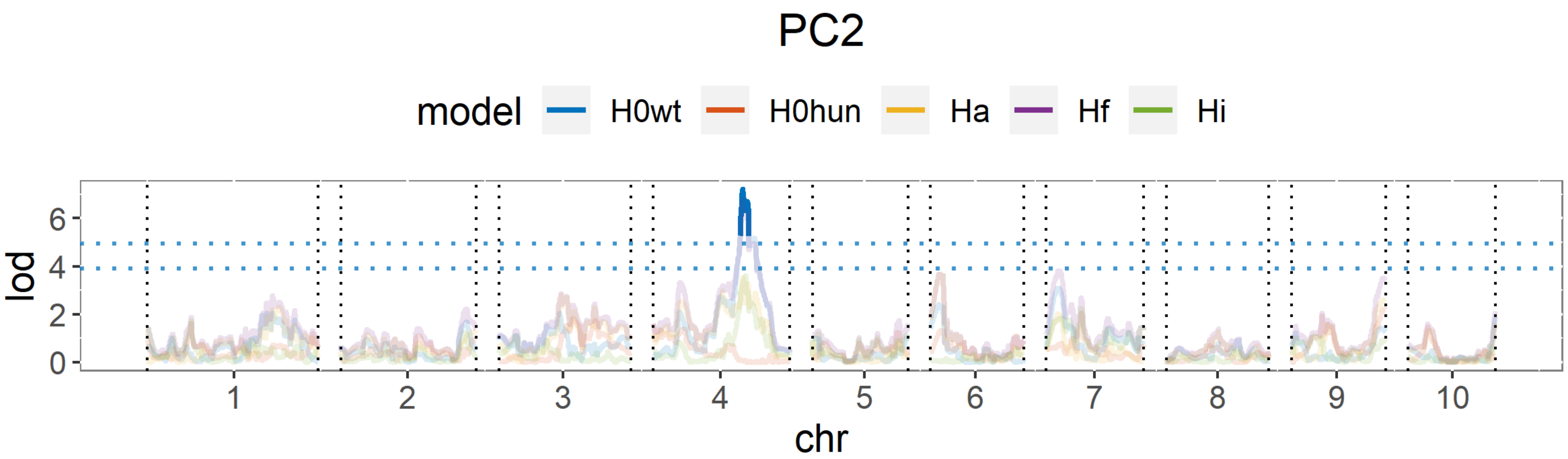
PC2
model
H0wt
H0hun
Ha
Hf
Hi
lod
0
2
4
6
chr
1
2
3
4
5
6
7
8
9
10

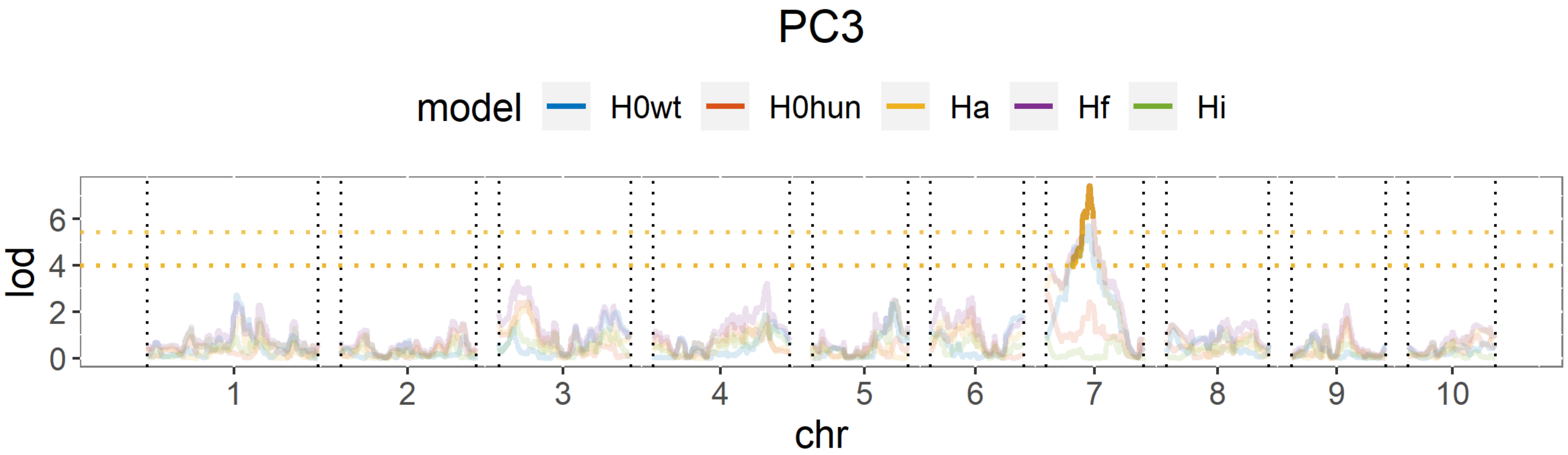

PC3
model
H0wt
H0hun
Ha
Hf
Hi
lod
6
4
2
0
1
2
3
4
5
6
7
8
9
10
chr

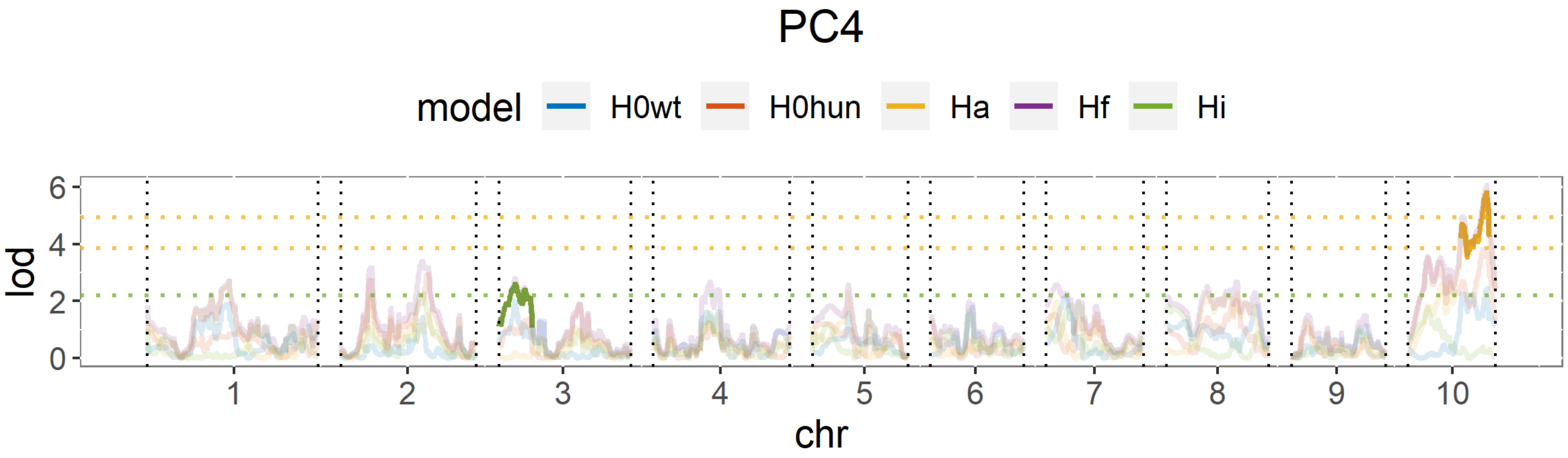
PC4
model
H0wt
H0hun
Ha
Hf
Hi
lod
0
2
4
6
chr
1
2
3
4
5
6
7
8
9
10

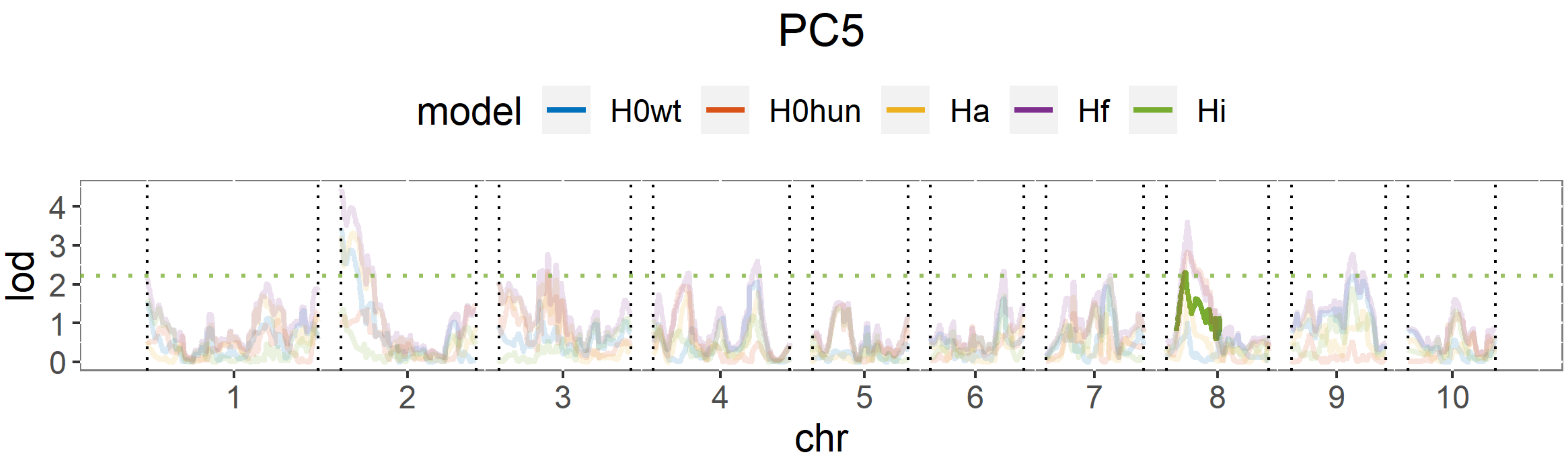

PC5
model
H0wt
H0hun
Ha
Hf
Hi
lod
0
1
2
3
4
1
2
3
4
5
6
7
8
9
10
chr

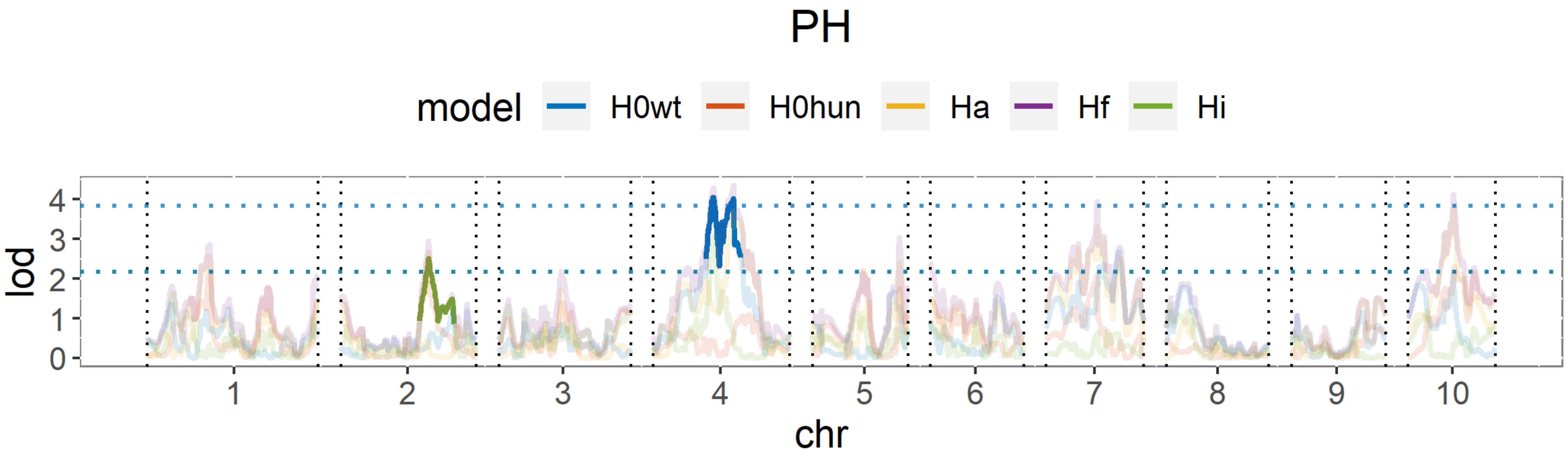
PH
model
H0wt
H0hun
Ha
Hf
Hi
lod
4
3
2
1
0
1
2
3
4
5
6
7
8
9
10
chr

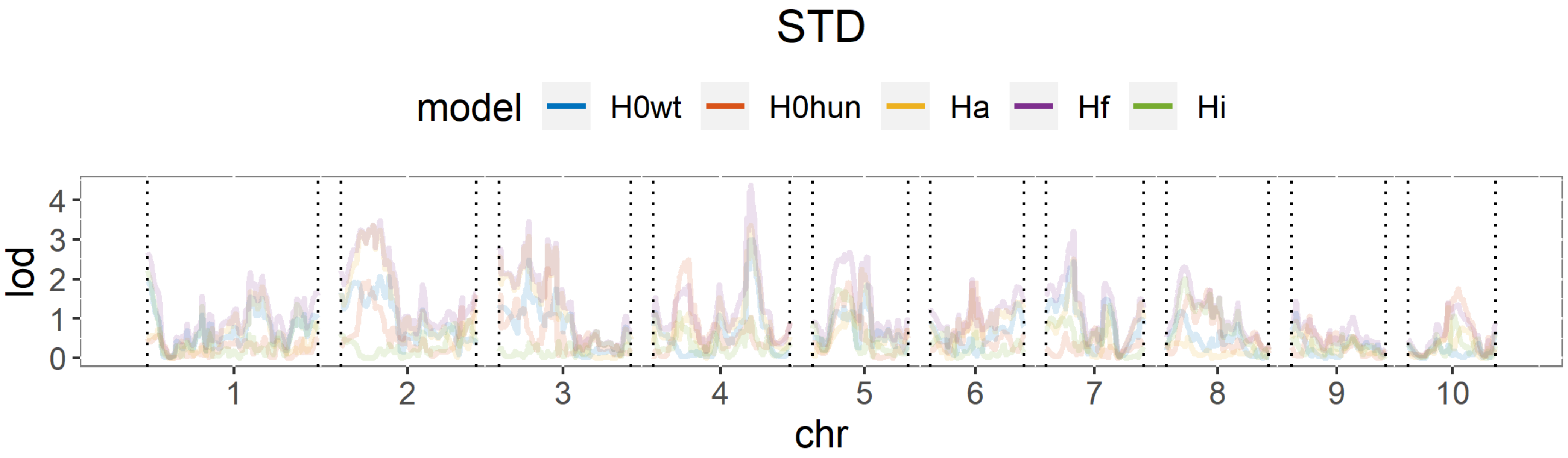

STD
model
H0wt
H0hun
Ha
Hf
Hi
lod
0
1
2
3
4
1
2
3
4
5
6
7
8
9
10
chr

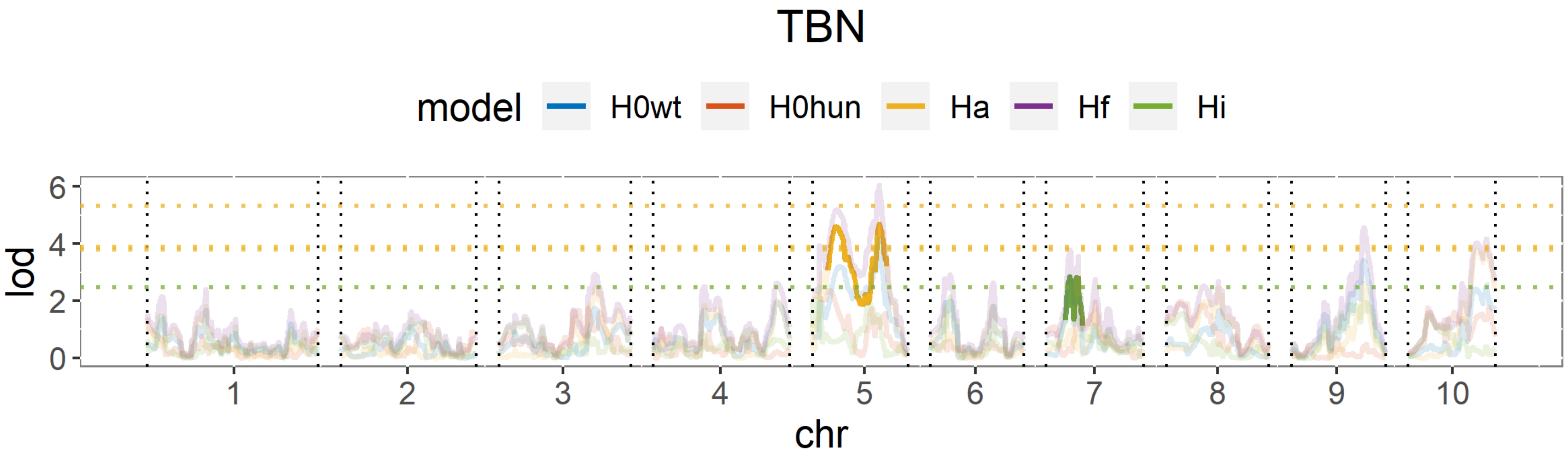

TBN
model
H0wt
H0hun
Ha
Hf
Hi
lod
0
2
4
6
chr
1
2
3
4
5
6
7
8
9
10

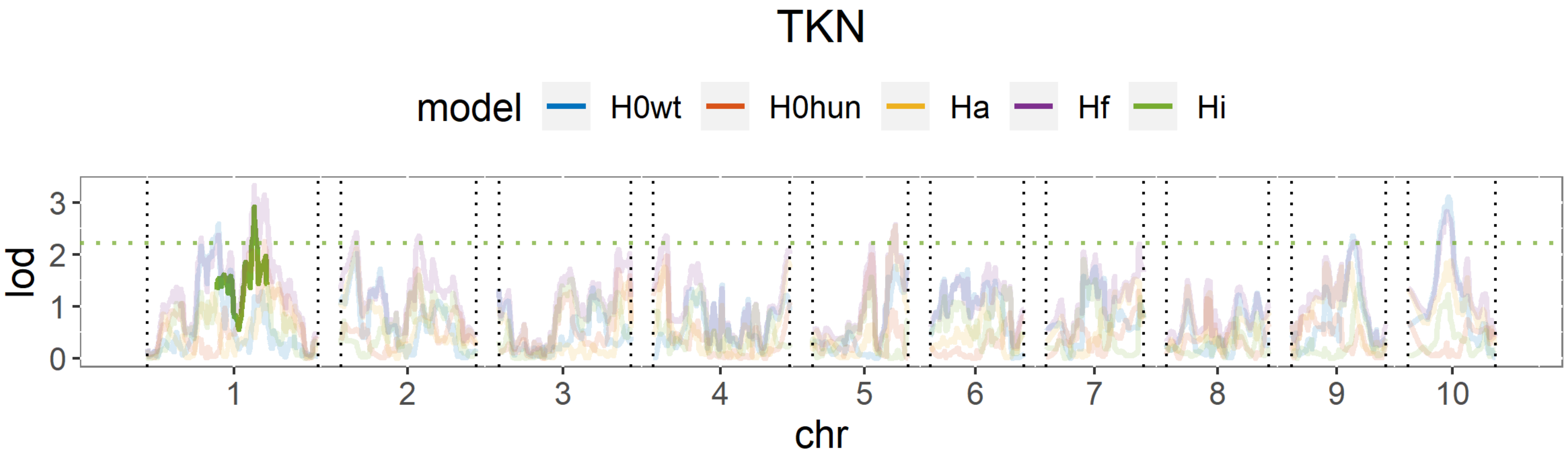

TKN
model
H0wt
H0hun
Ha
Hf
Hi
lod
0
1
2
3
1
2
3
4
5
6
7
8
9
10
chr

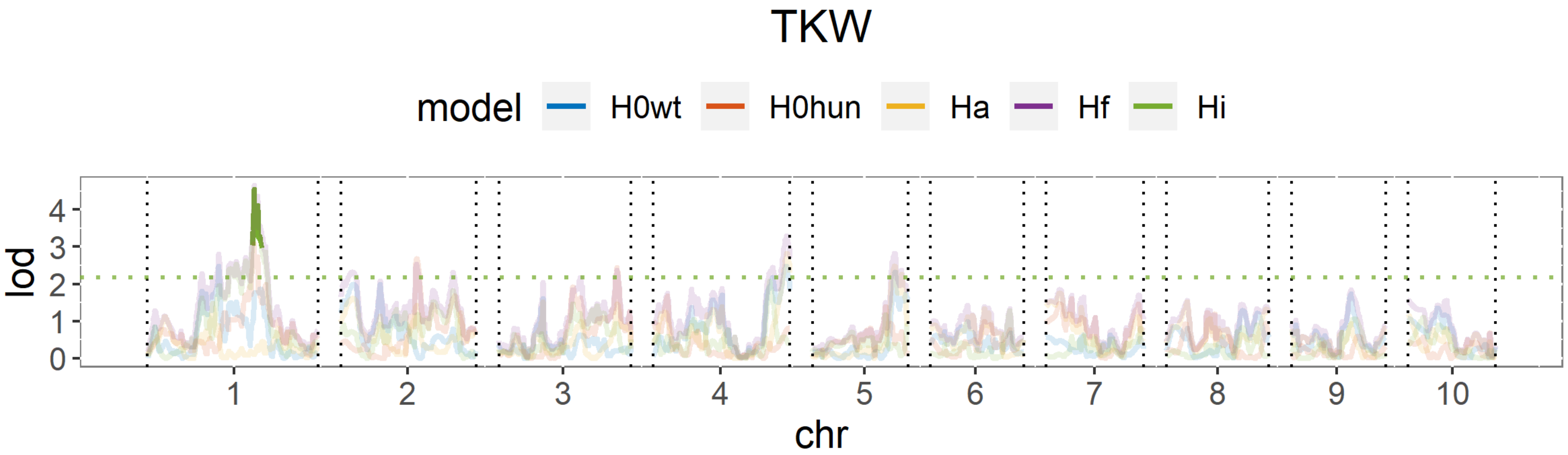

TKW
model
H0wt
H0hun
Ha
Hf
Hi
lod
0
1
2
3
4
chr
1
2
3
4
5
6
7
8
9
10

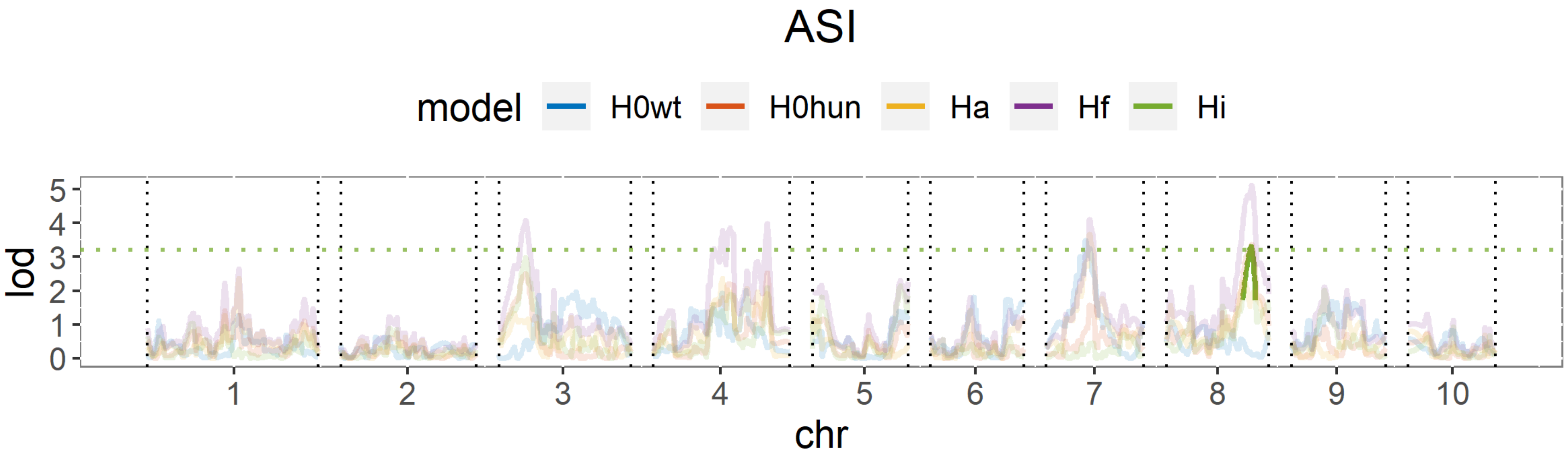

ASI
model
H0wt
H0hun
Ha
Hf
Hi
lod
5
4
3
2
1
0
1
2
3
4
5
6
7
8
9
10
chr

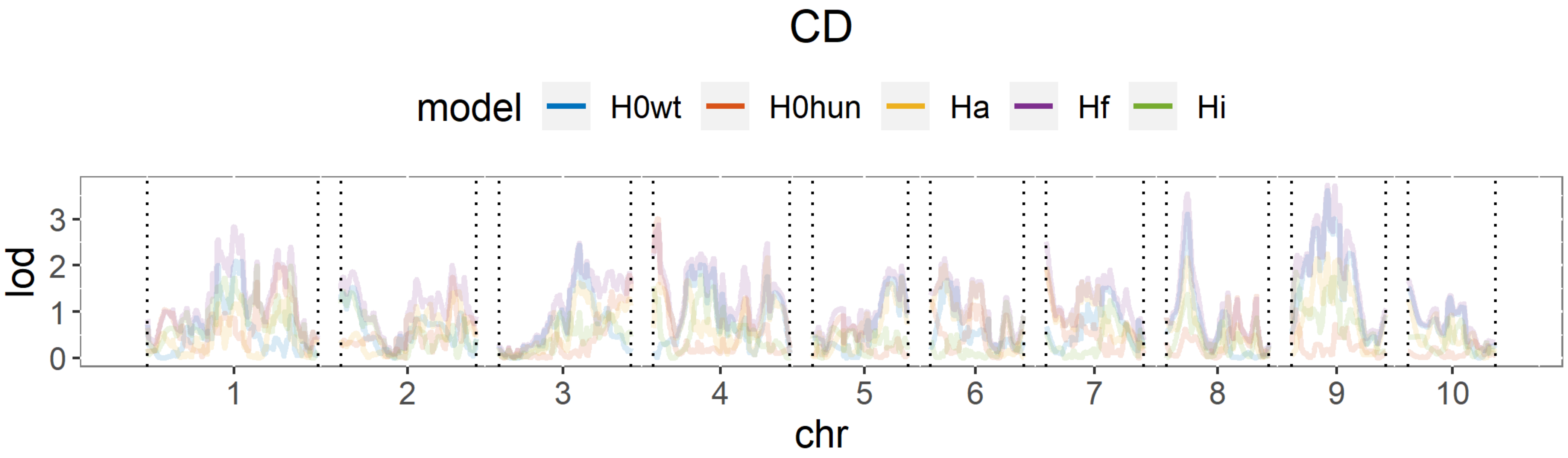

CD
model
H0wt
H0hun
Ha
Hf
Hi
lod
0
1
2
3
1
2
3
4
5
6
7
8
9
10
chr

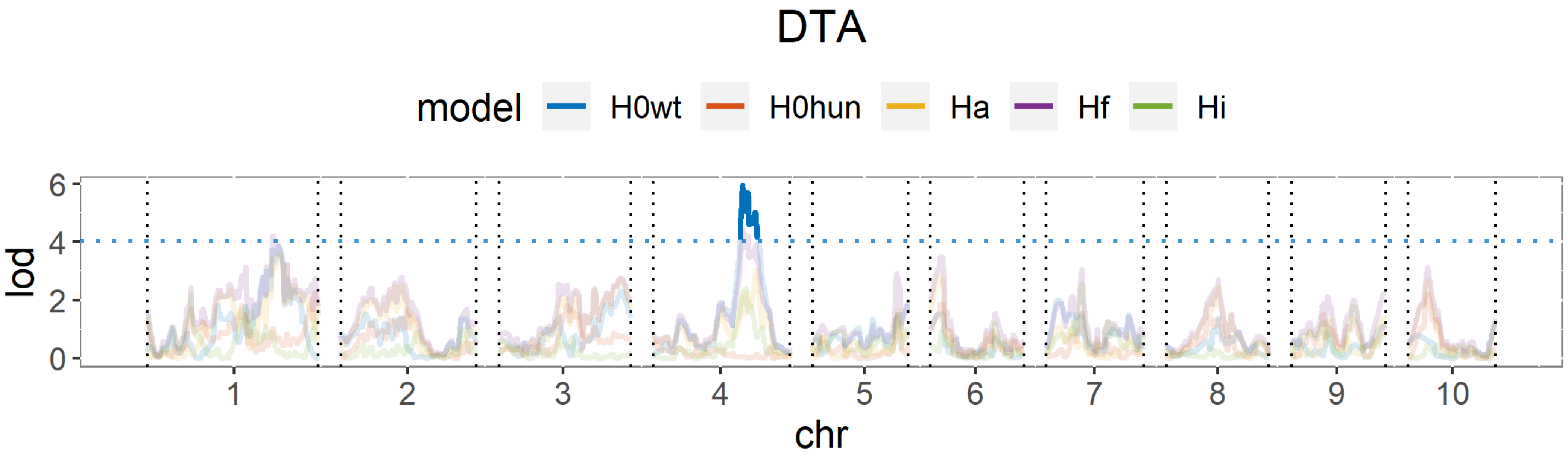
DTA
model
H0wt
H0hun
Ha
Hf
Hi
lod
0
2
4
6
1
2
3
4
5
6
7
8
9
10
chr

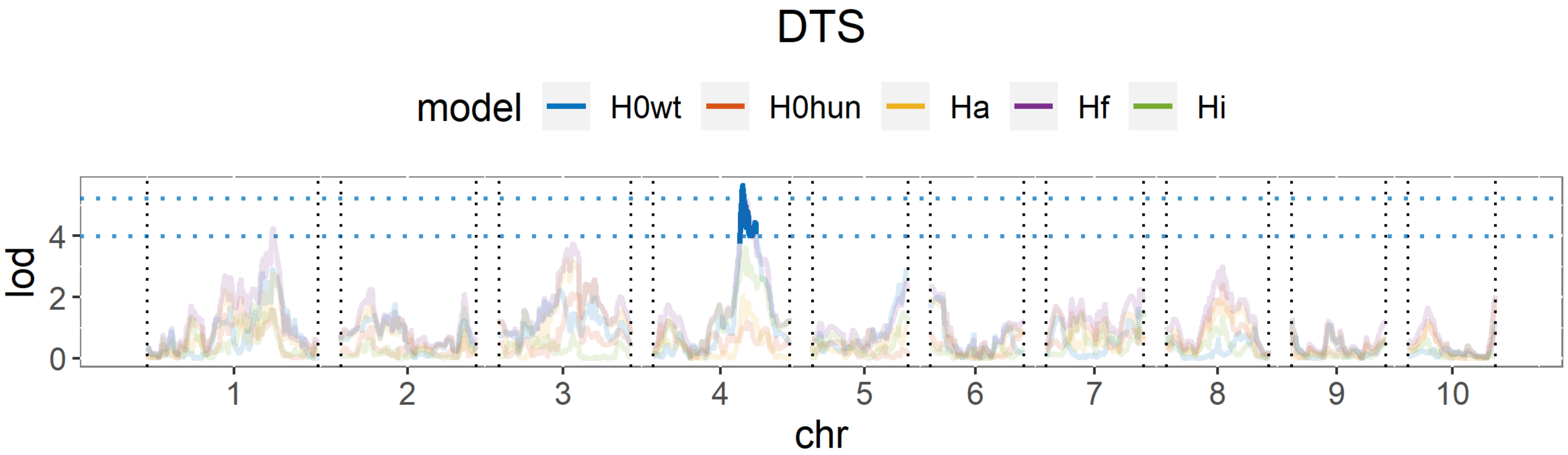

DTS
model
H0wt
H0hun
Ha
Hf
Hi
lod
0
2
4
1
2
3
4
5
6
7
8
9
10
chr

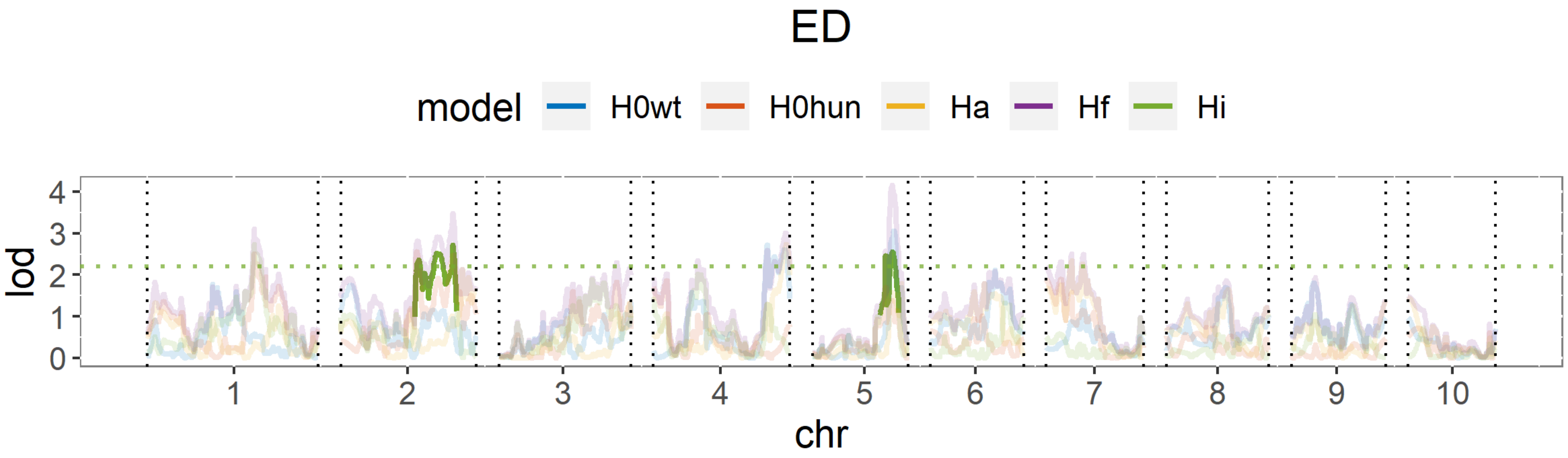

ED
model
H0wt
H0hun
Ha
Hf
Hi
lod
0
1
2
3
4
1
2
3
4
5
6
7
8
9
10
chr

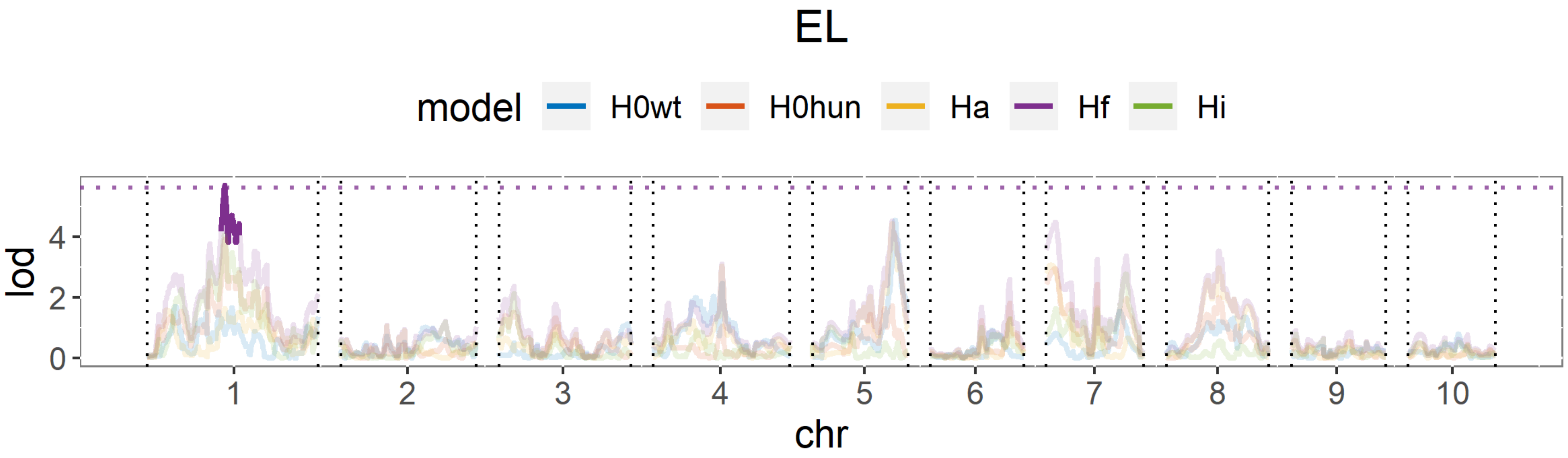
EL
model
H0wt
H0hun
Ha
Hf
Hi
lod
0
2
4
1
2
3
4
5
6
7
8
9
10
chr

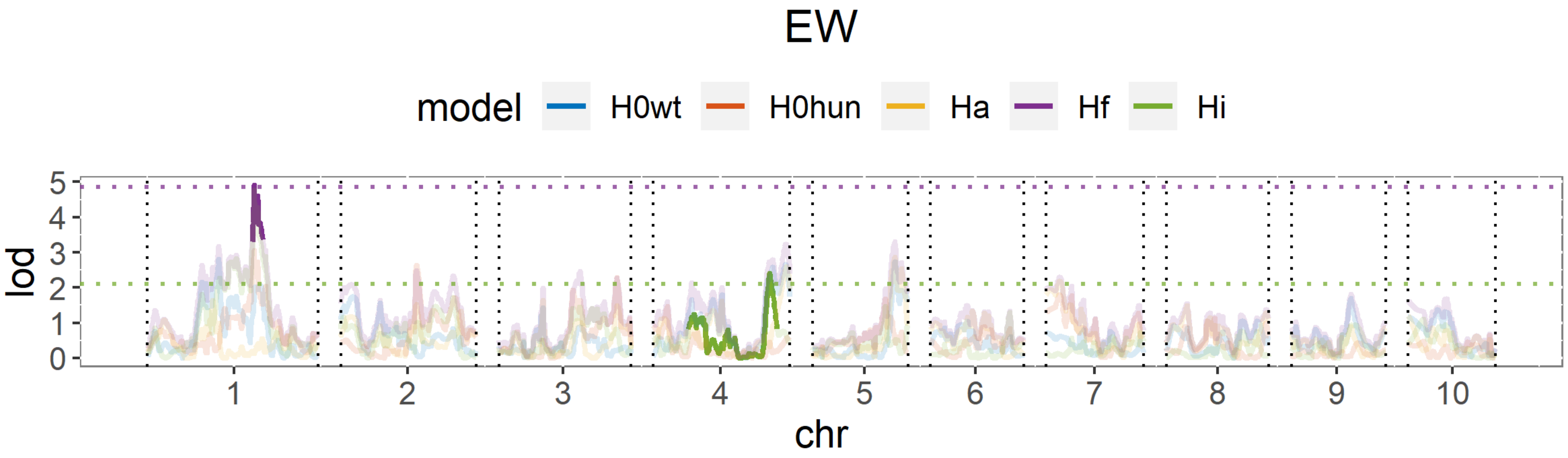

EW
model H0wt H0hun Ha Hf Hi
lod
0
1
2
3
4
5
chr
1 2 3 4 5 6 7 8 9 10

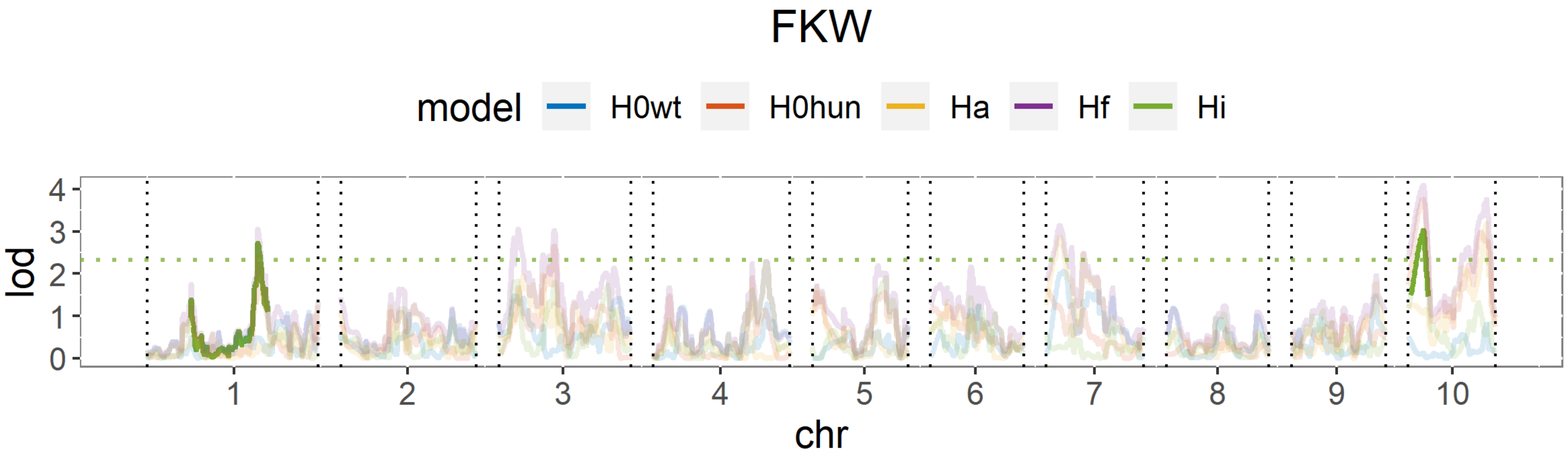
FKW
model
H0wt
H0hun
Ha
Hf
Hi
lod
4
3
2
1
0
1
2
3
4
5
6
7
8
9
10
chr

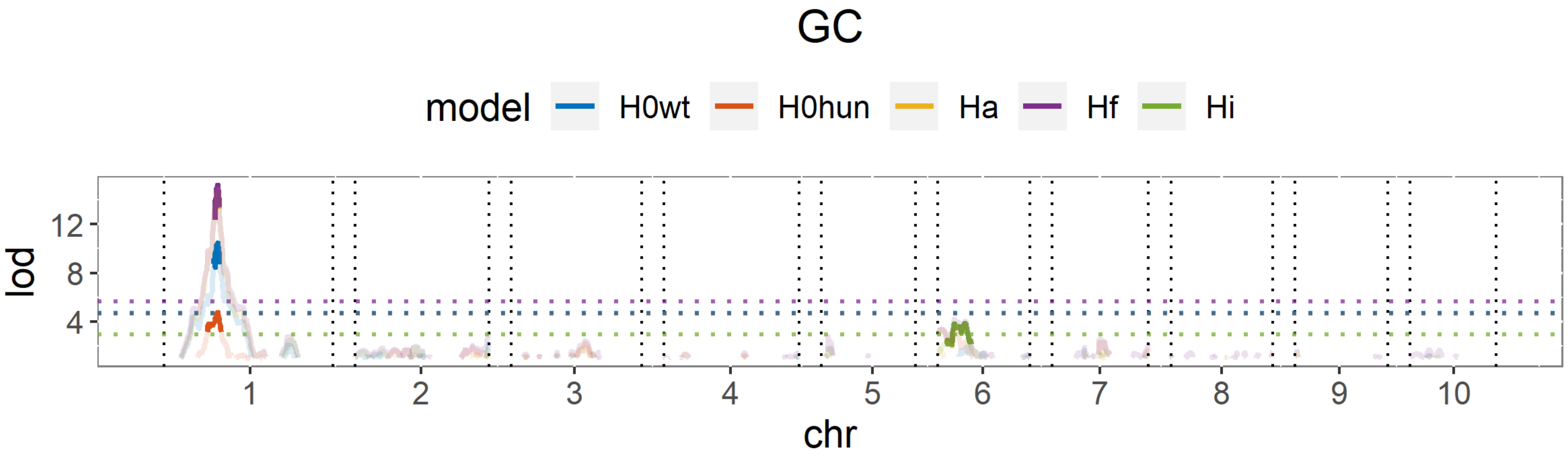

GC
model
H0wt
H0hun
Ha
Hf
Hi
lod
12
8
4
1
2
3
4
5
6
7
8
9
10
chr
