# Supplementary material for: The genetic architecture of host response reveals the importance of arbuscular mycorrhizae to maize cultivation
Source: eLife. 2020 Nov 19;9:e61701. doi: 10.7554/eLife.61701 (PMC7676867; doi:10.7554/eLife.61701)
Supplement: Figure 4—source data 1. — Effect plots for major QTL detected in the analysis across susceptible (AMF-S) and resistant (AMF-R) families. The title of the plot gives the marker nearest to the LOD peak, and the color of the line represents the genotype at the QTL. [file elife-61701-fig4-data1.pdf]

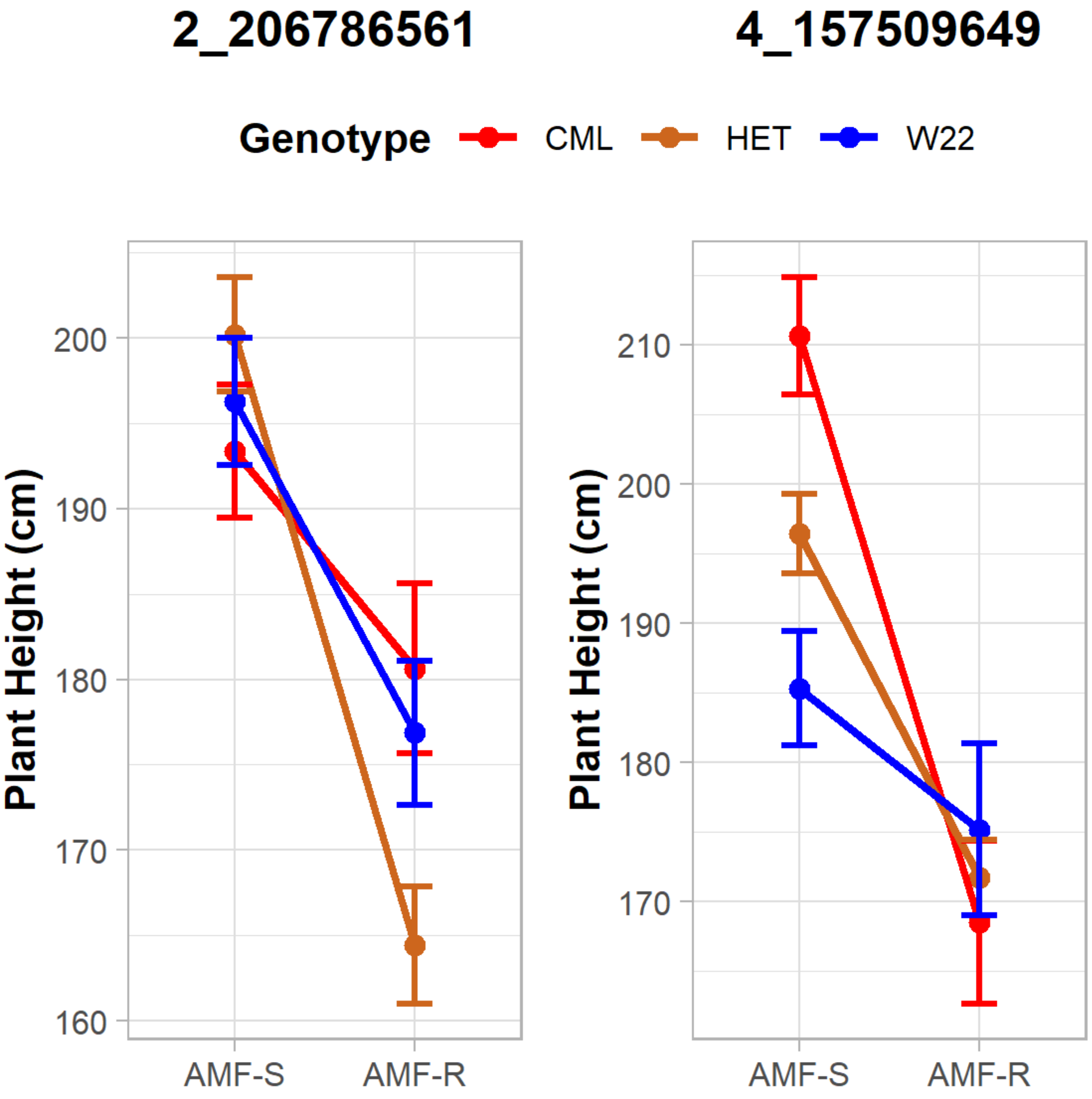

2_206786561
4_157509649
Genotype
CML
HET
W22
Plant Height (cm)
160
170
180
190
200
210
AMF-S
AMF-R

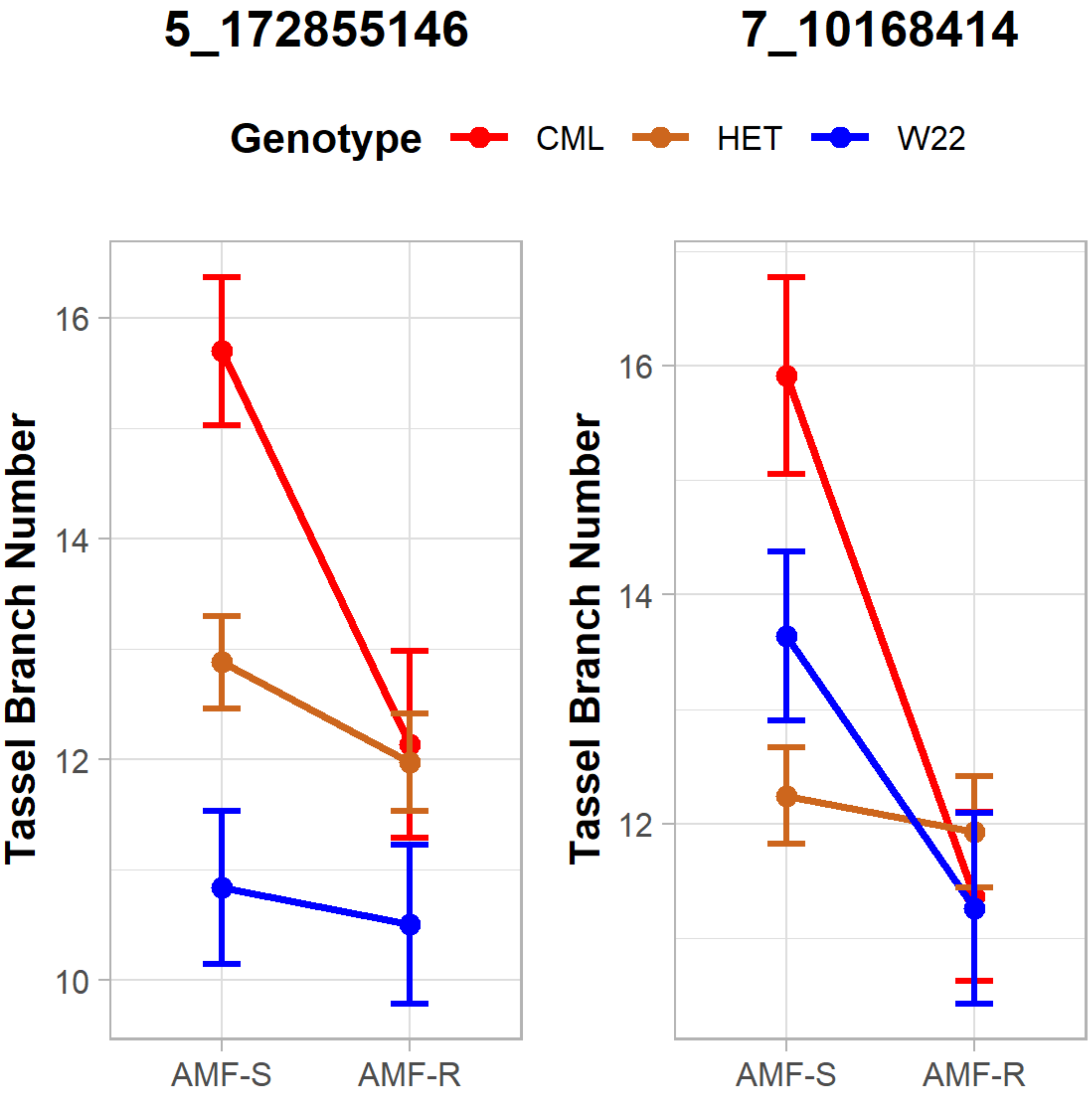

5_172855146
7_10168414
Genotype
CML
HET
W22
Tassel Branch Number
16
14
12
10
AMF-S
AMF-R
Tassel Branch Number
16
14
12
AMF-S
AMF-R

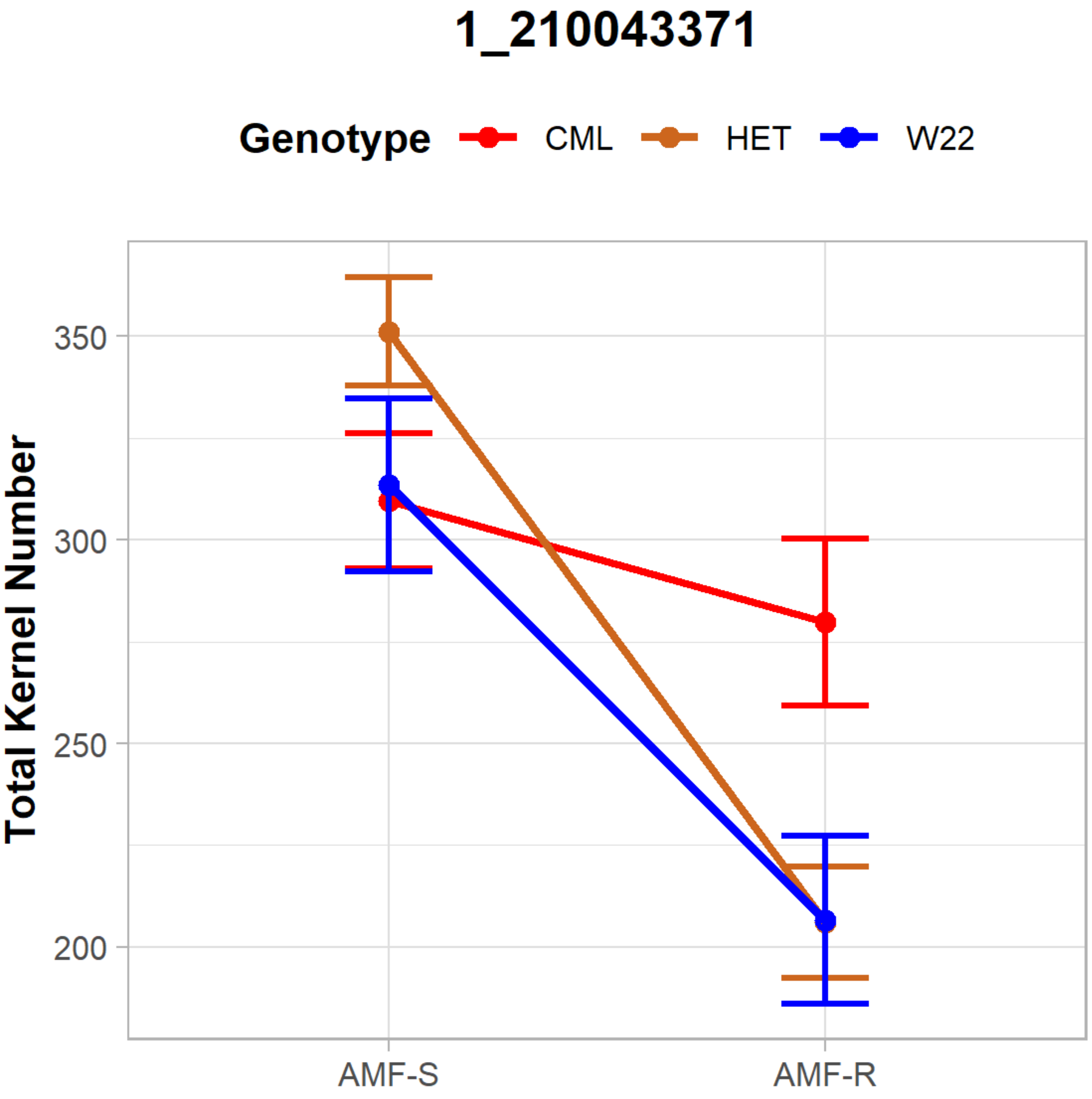

1_210043371
Genotype
CML
HET
W22
Total Kernel Number
350
300
250
200
AMF-S
AMF-R

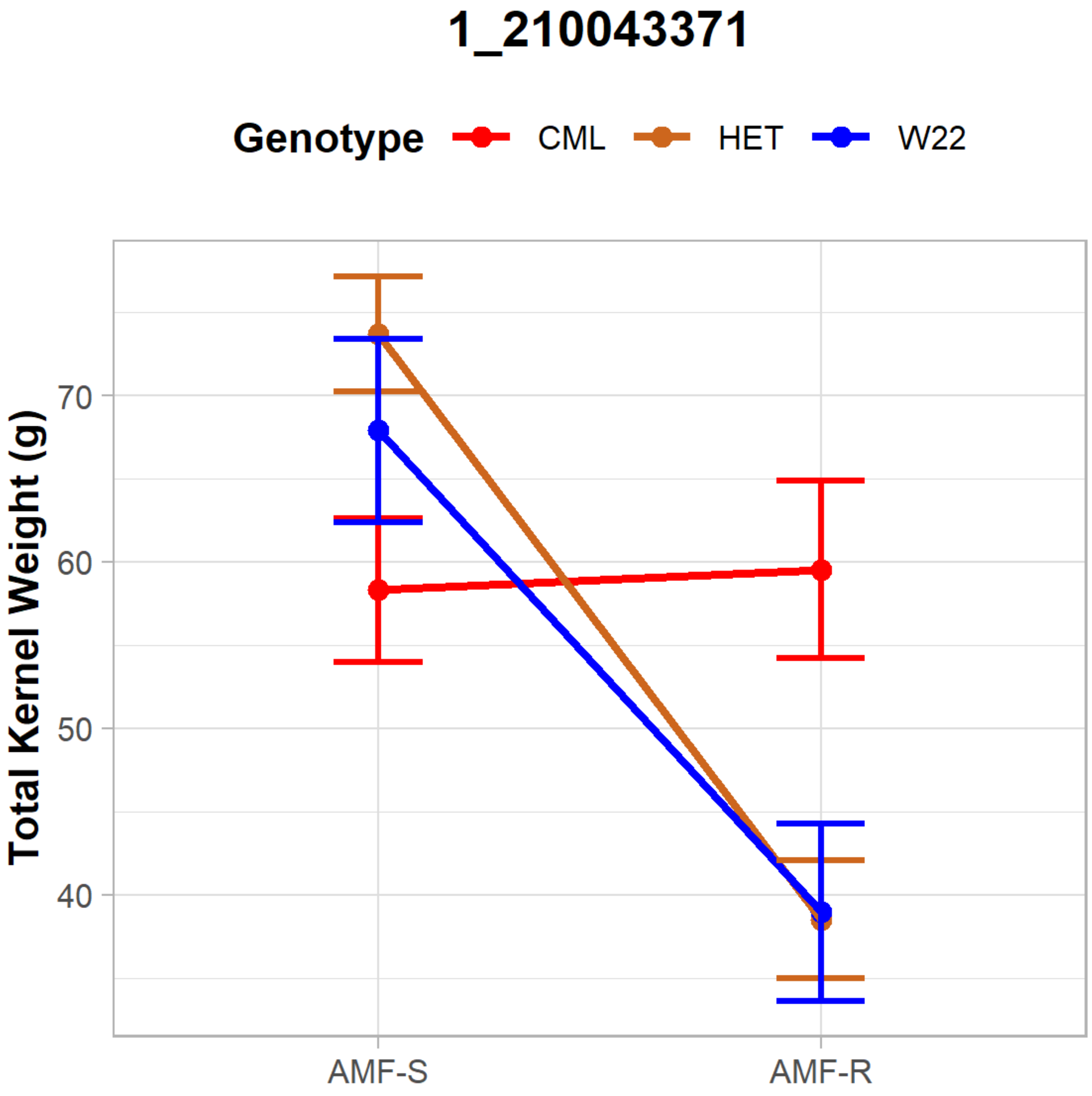
1_210043371
Genotype
CML
HET
W22
Total Kernel Weight (g)
70
60
50
40
AMF-S
AMF-R

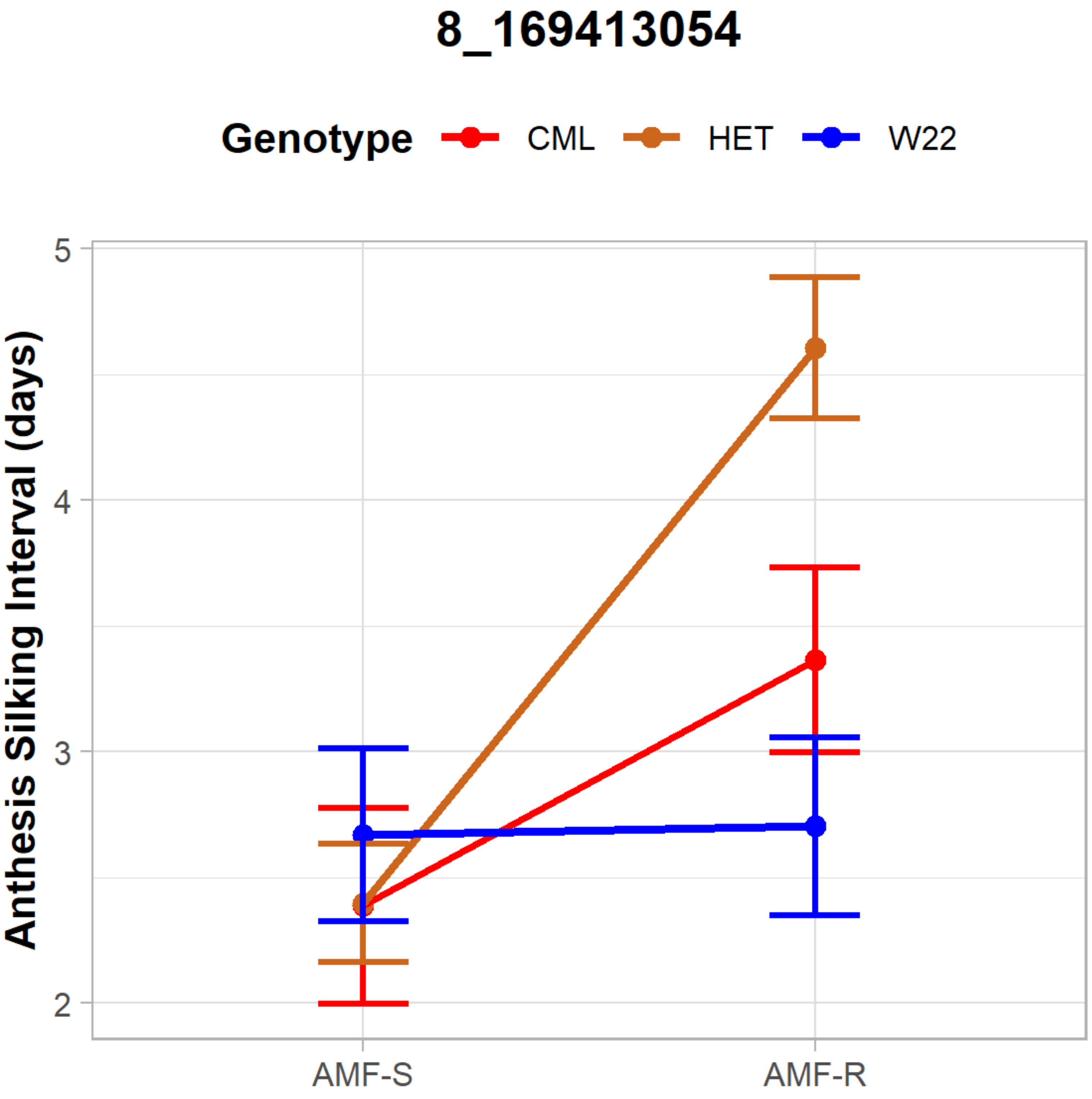
8_169413054
Genotype
CML
HET
W22
Anthesis Silking Interval (days)
5
4
3
2
AMF-S
AMF-R

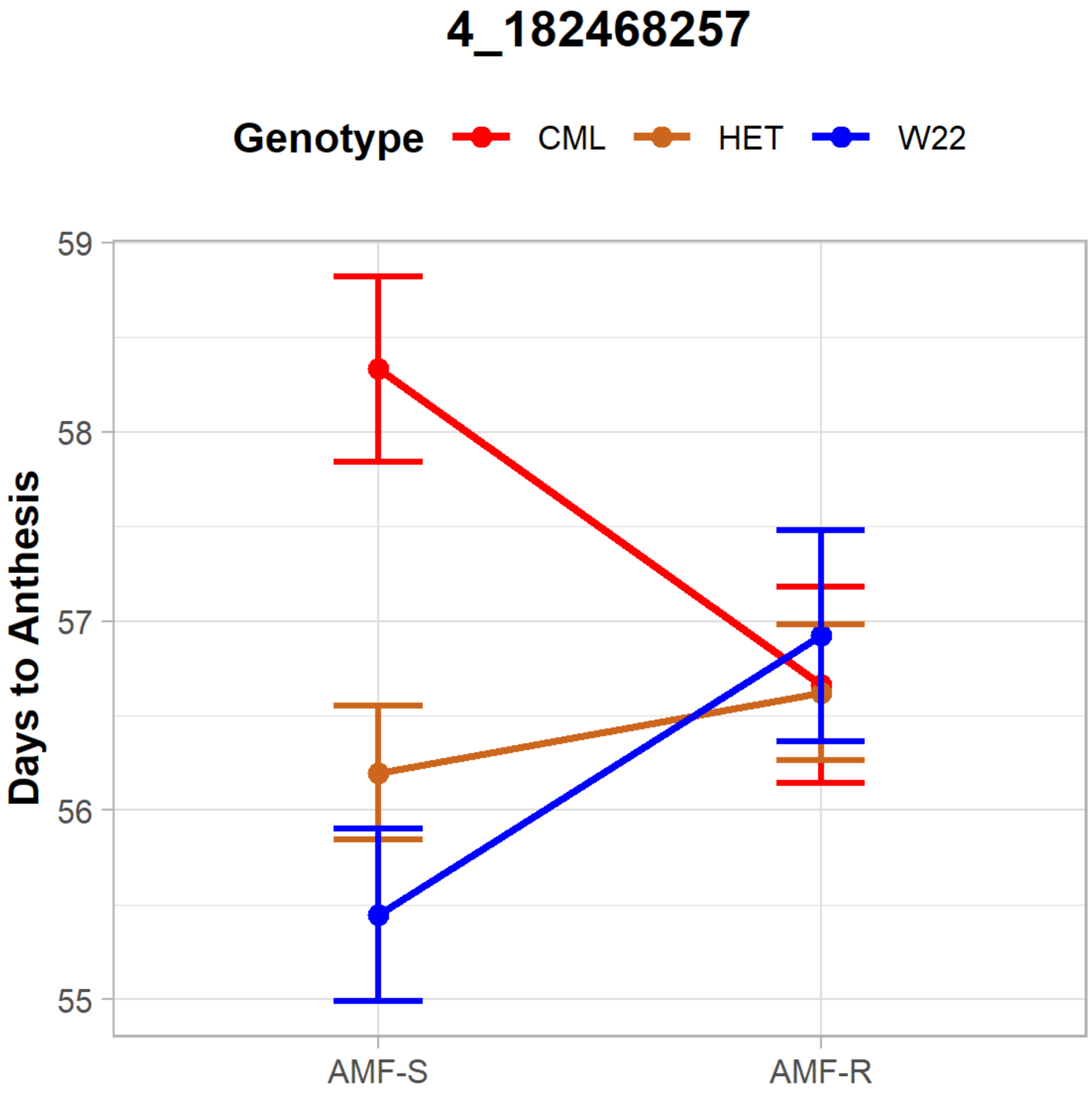
4_182468257
Genotype
CML
HET
W22
Days to Anthesis
59
58
57
56
55
AMF-S
AMF-R

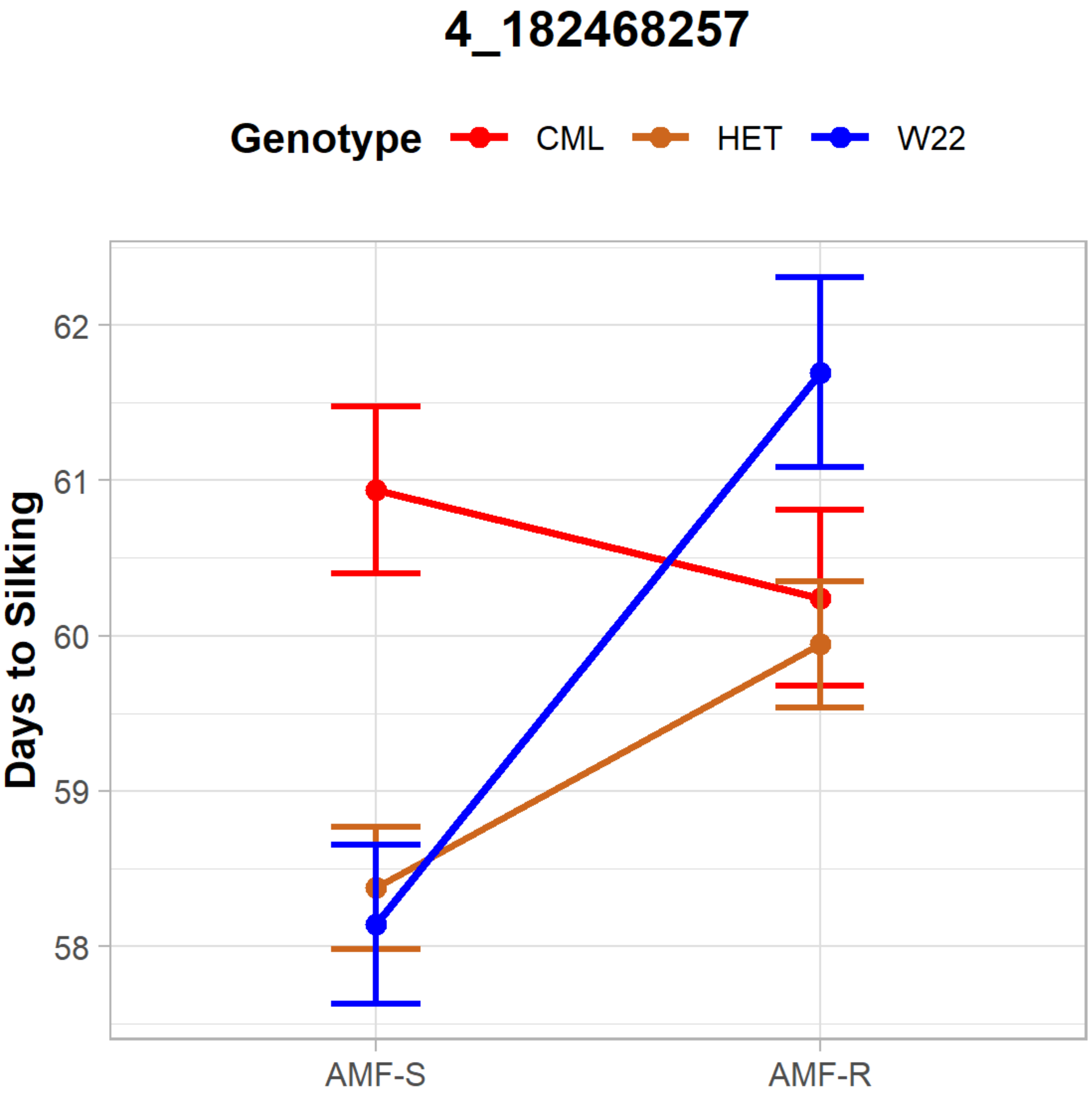
4_182468257
Genotype
CML
HET
W22
Days to Silking
62
61
60
59
58
AMF-S
AMF-R

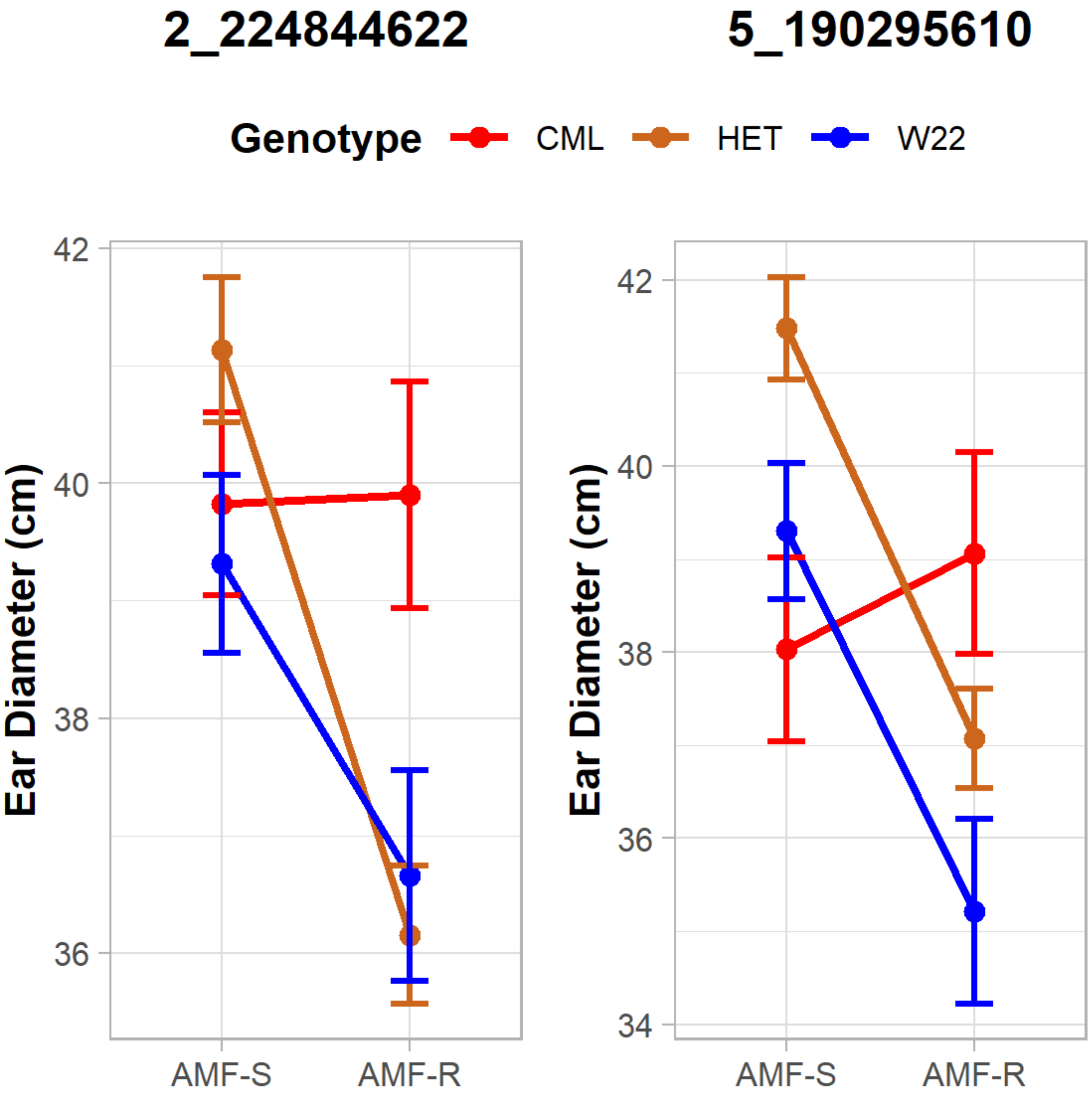

2_224844622
5_190295610
Genotype
CML
HET
W22
Ear Diameter (cm)
42
40
38
36
34
AMF-S
AMF-R

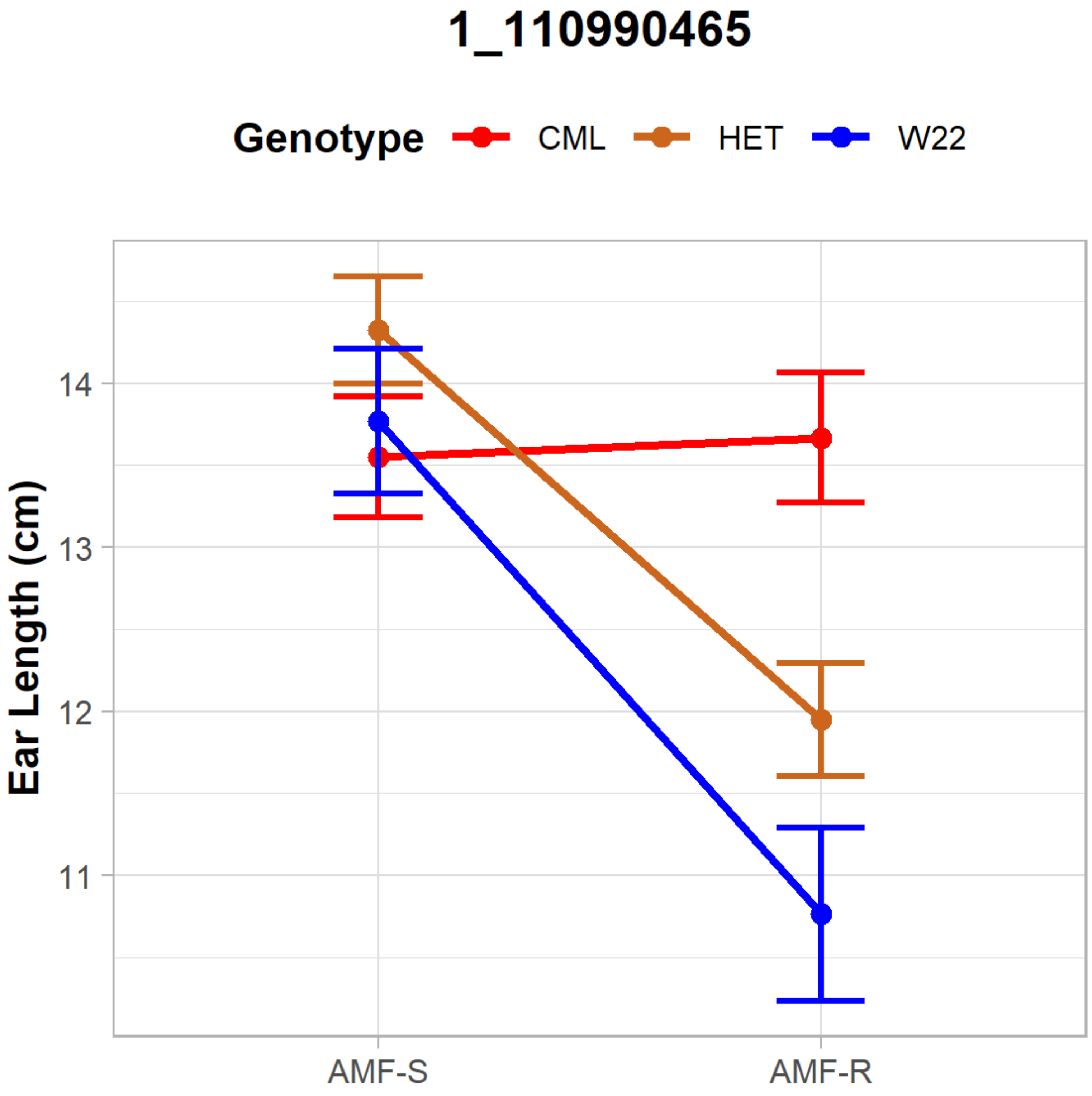

1_110990465
Genotype
CML
HET
W22
Ear Length (cm)
14
13
12
11
AMF-S
AMF-R

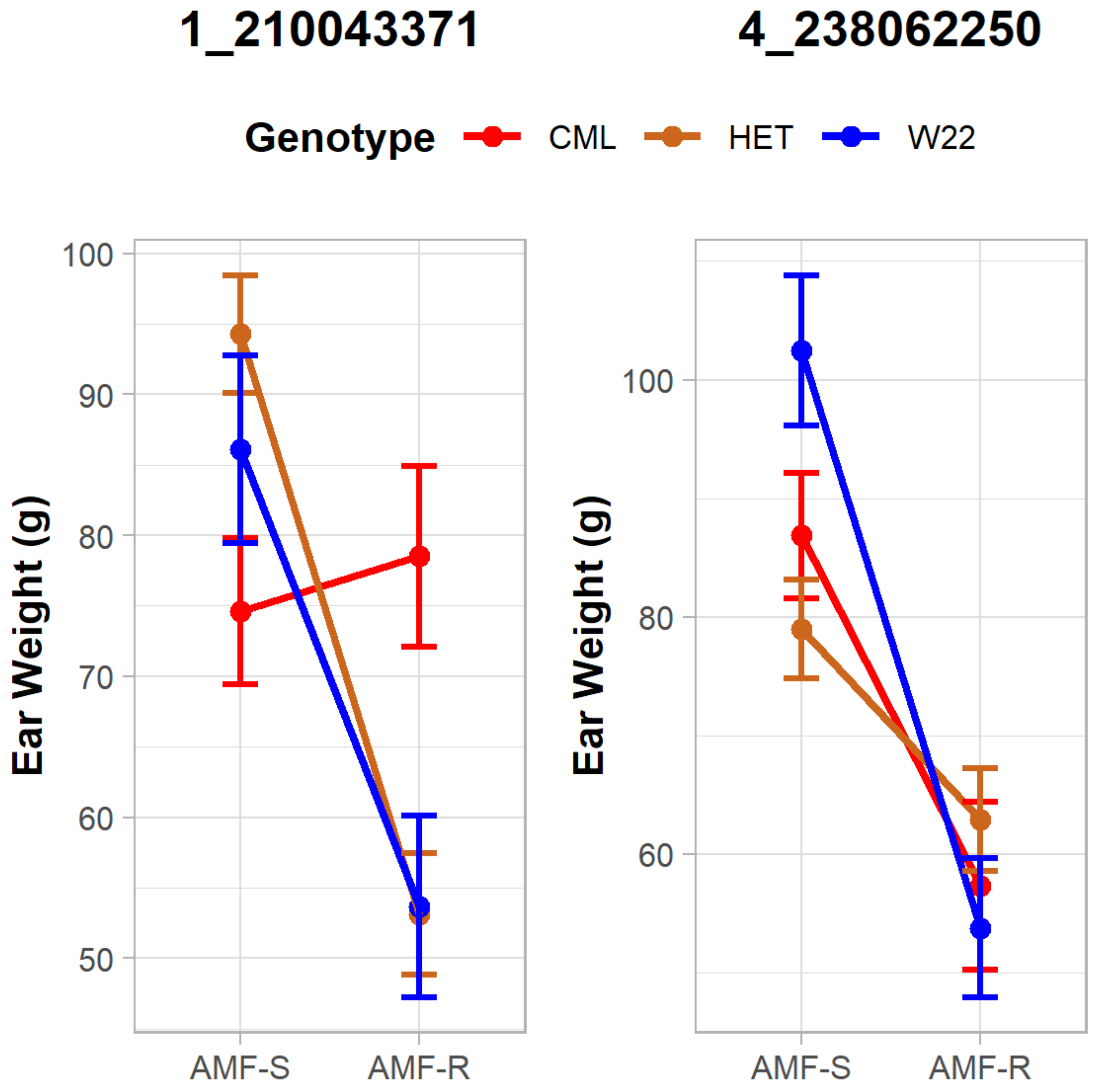
1_210043371
4_238062250
Genotype
CML
HET
W22
Ear Weight (g)
100
90
80
70
60
50
AMF-S
AMF-R
Ear Weight (g)
100
80
60
AMF-S
AMF-R

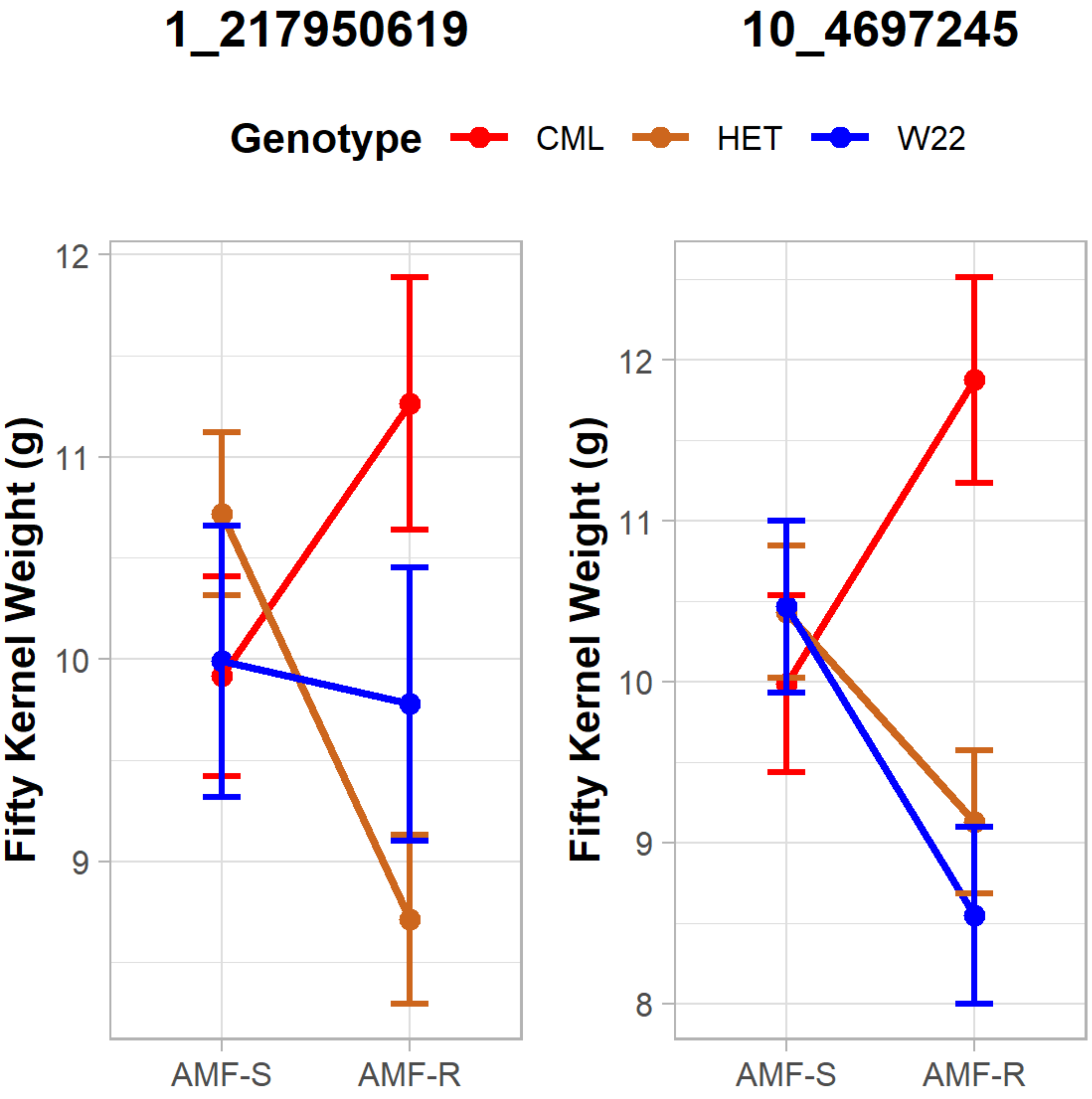

1_217950619
10_4697245
Genotype
CML
HET
W22
Fifty Kernel Weight (g)
12
11
10
9
AMF-S
AMF-R
Fifty Kernel Weight (g)
12
11
10
9
8
AMF-S
AMF-R

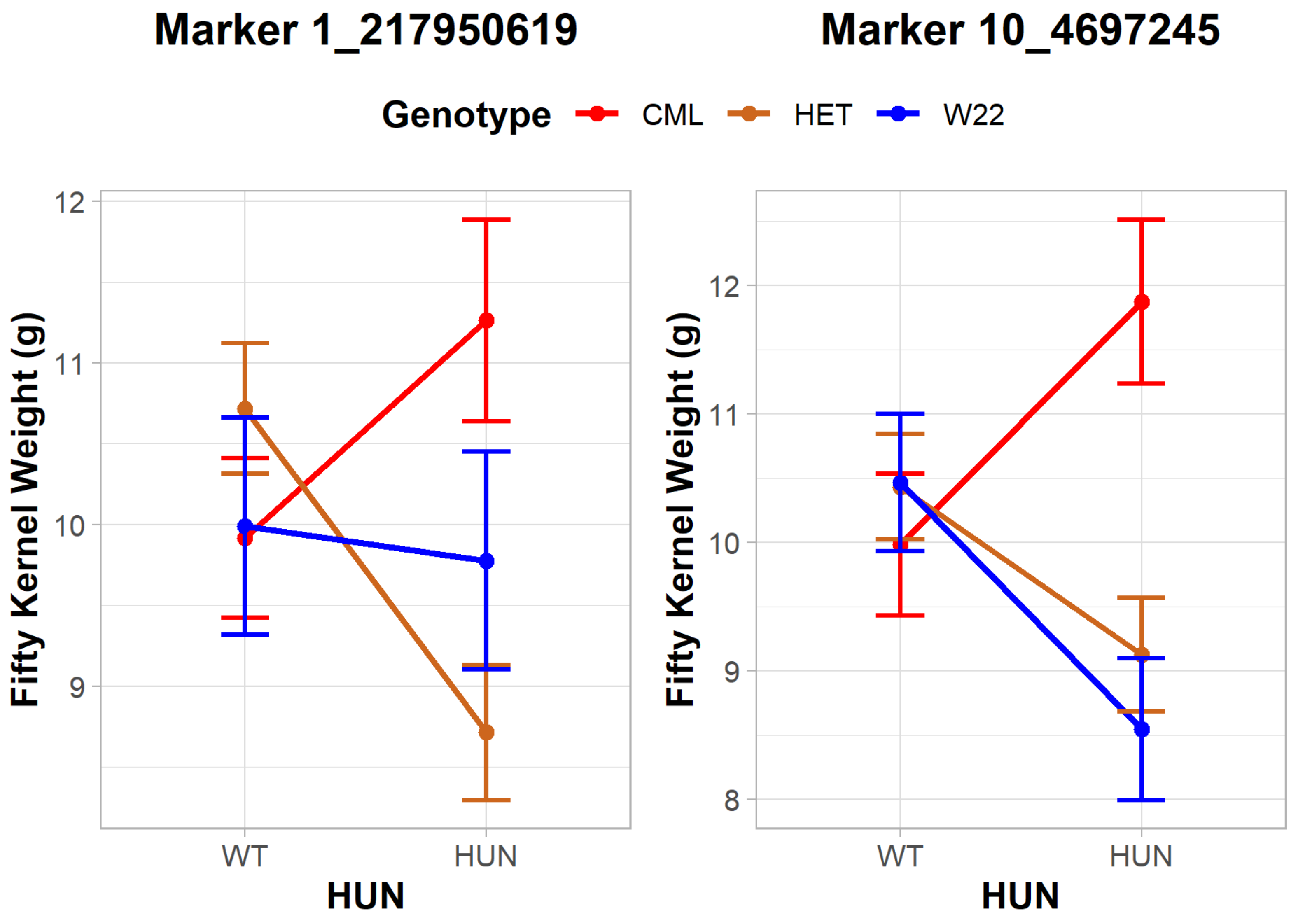
Marker 1_217950619
Marker 10_4697245
Genotype
CML
HET
W22
Fifty Kernel Weight (g)
12
11
10
9
8
WT
HUN
HUN

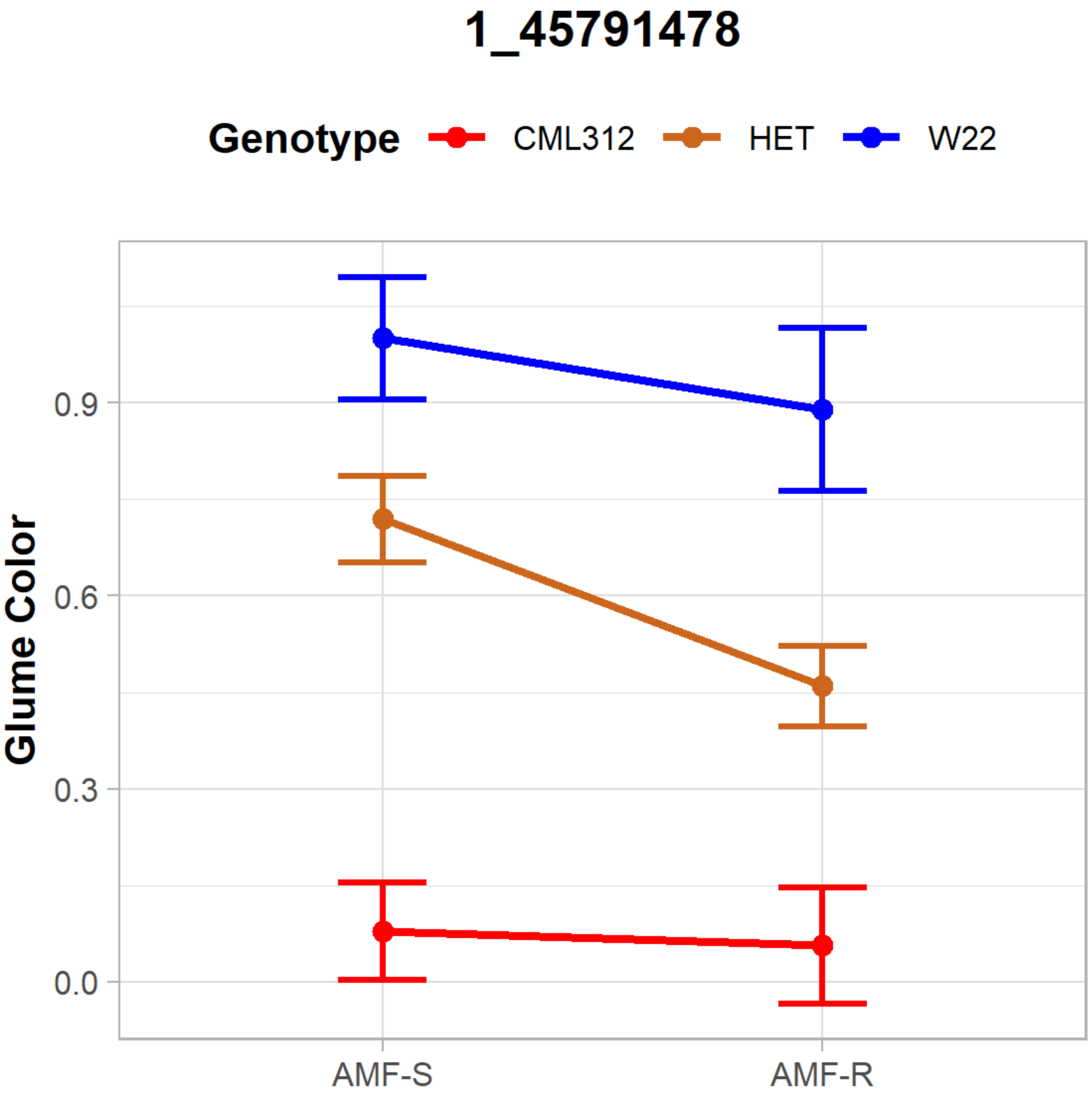

1_45791478
Genotype
CML312
HET
W22
Glume Color
0.9
0.6
0.3
0.0
AMF-S
AMF-R

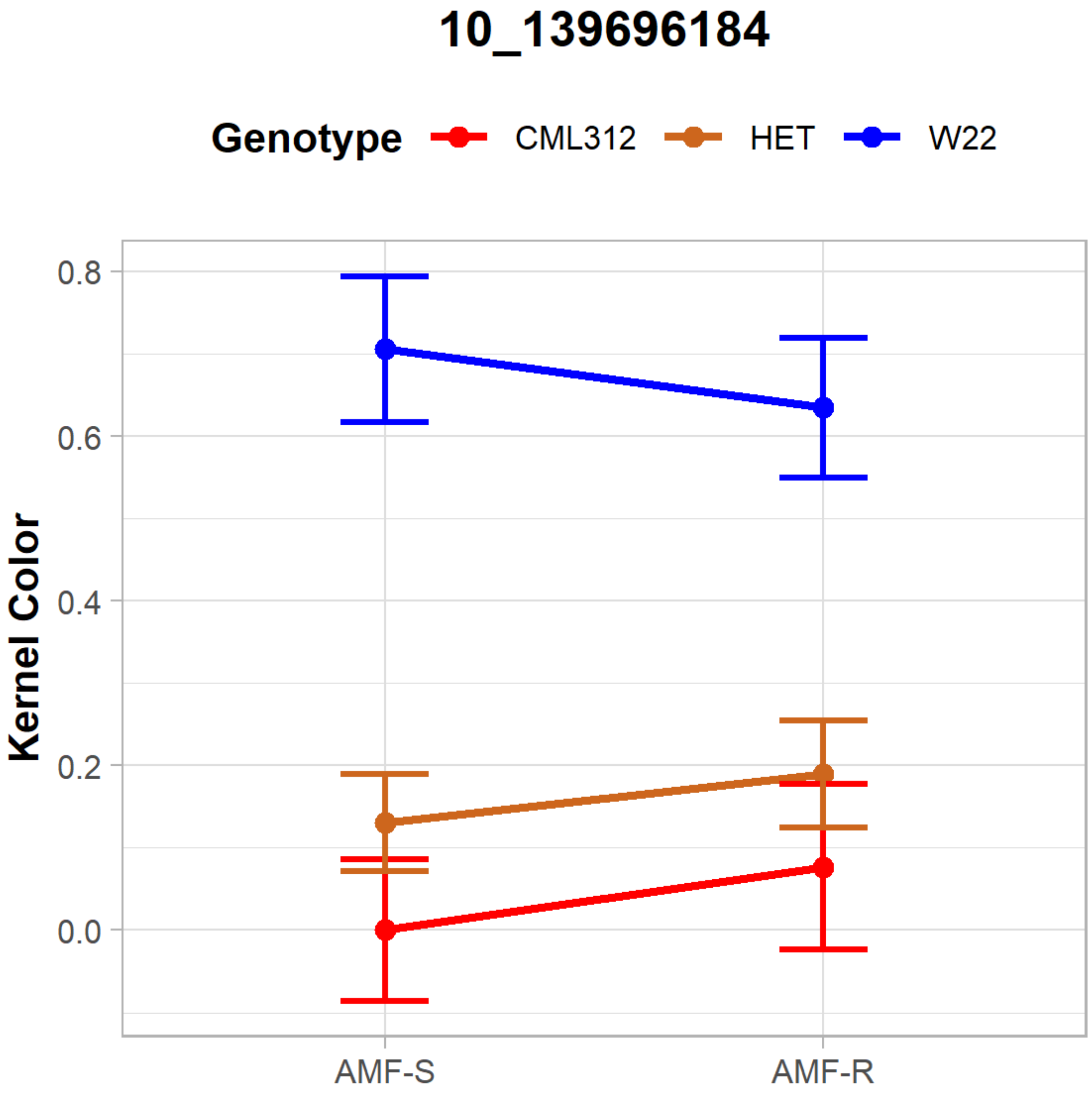
10_139696184
Genotype
CML312
HET
W22
Kernel Color
0.8
0.6
0.4
0.2
0.0
AMF-S
AMF-R

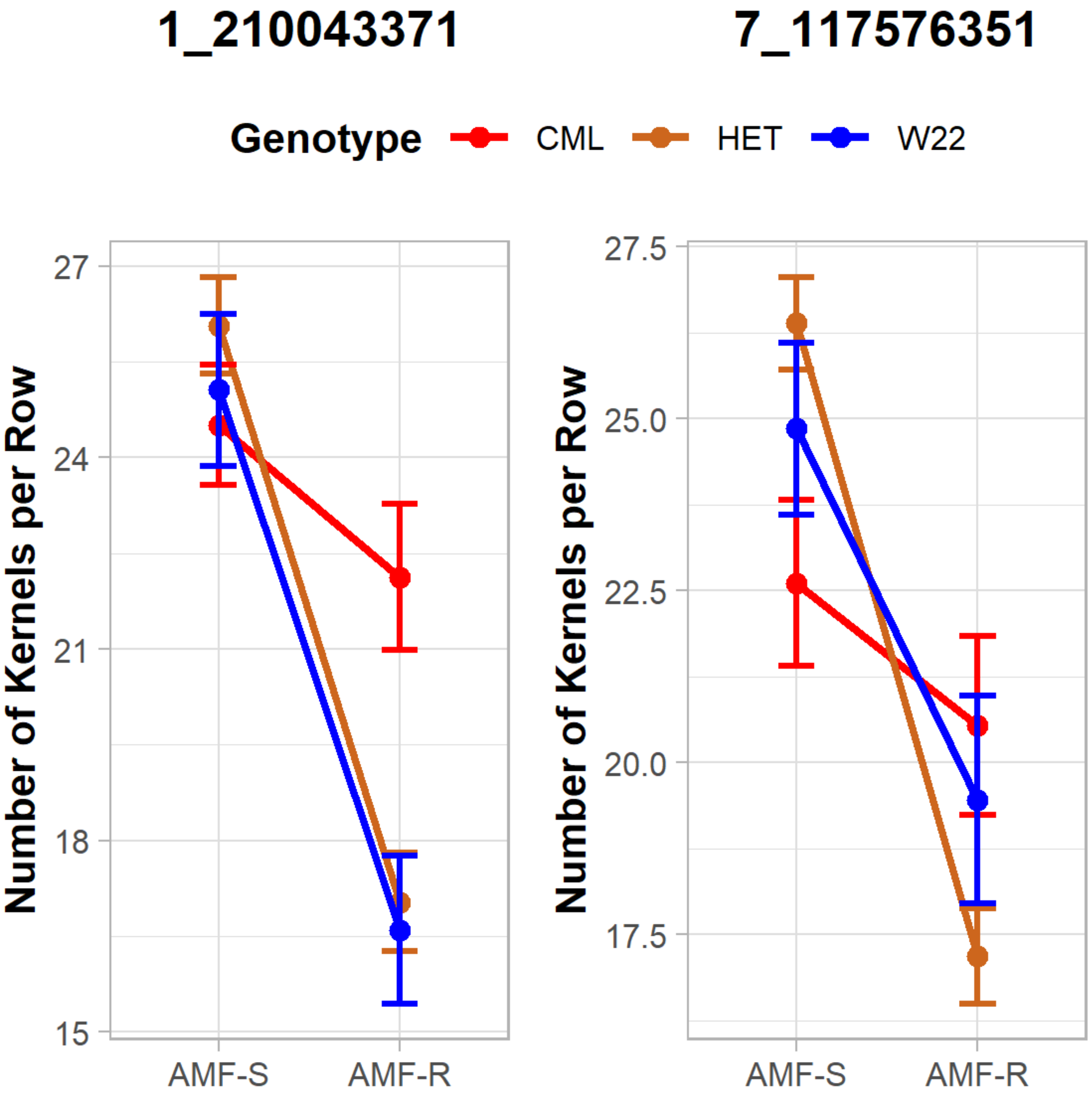

1_210043371
7_117576351
Genotype
CML
HET
W22
Number of Kernels per Row
27
24
21
18
15
27.5
25.0
22.5
20.0
17.5
AMF-S
AMF-R

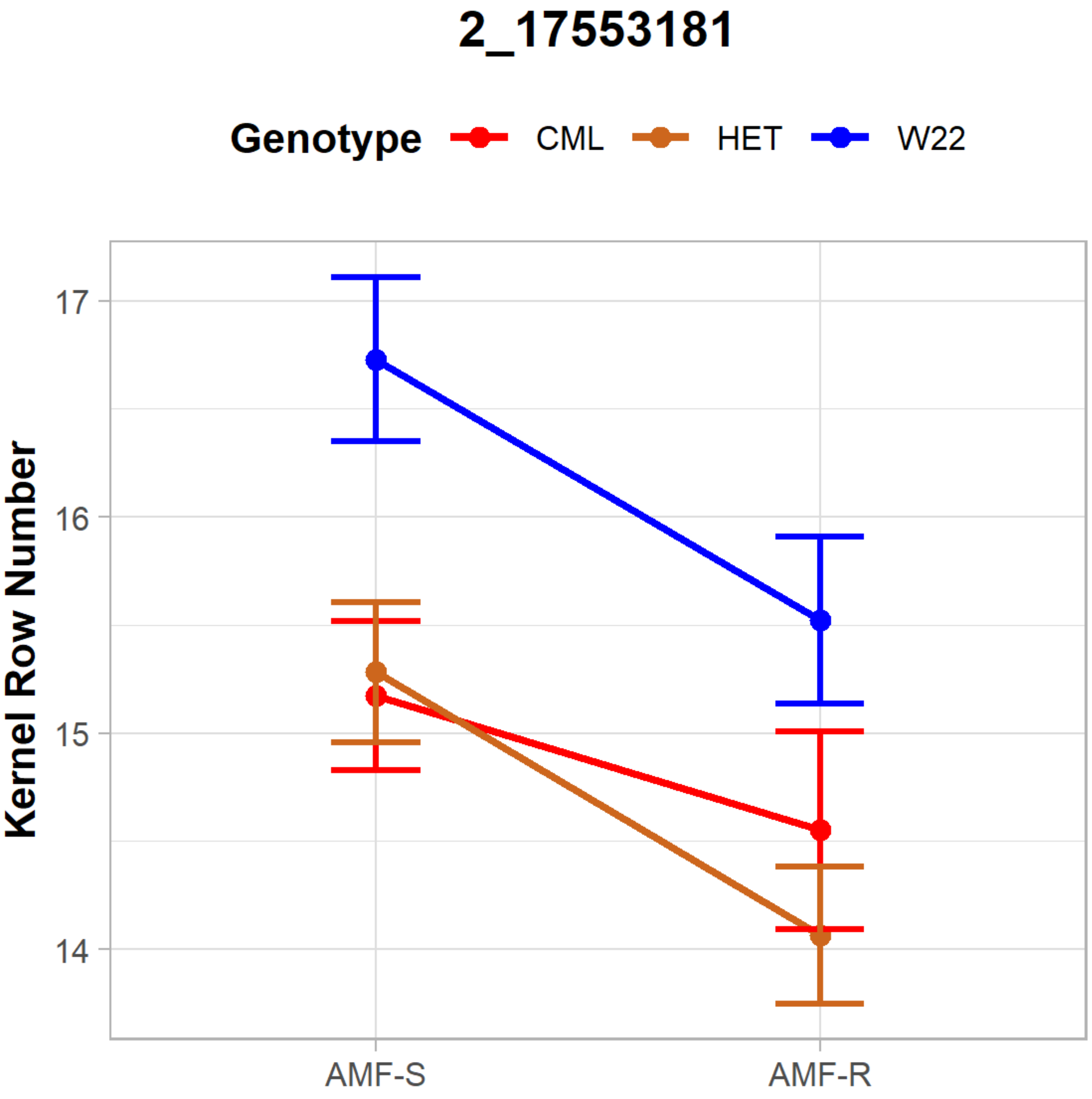

2_17553181
Genotype
CML
HET
W22
Kernel Row Number
17
16
15
14
AMF-S
AMF-R

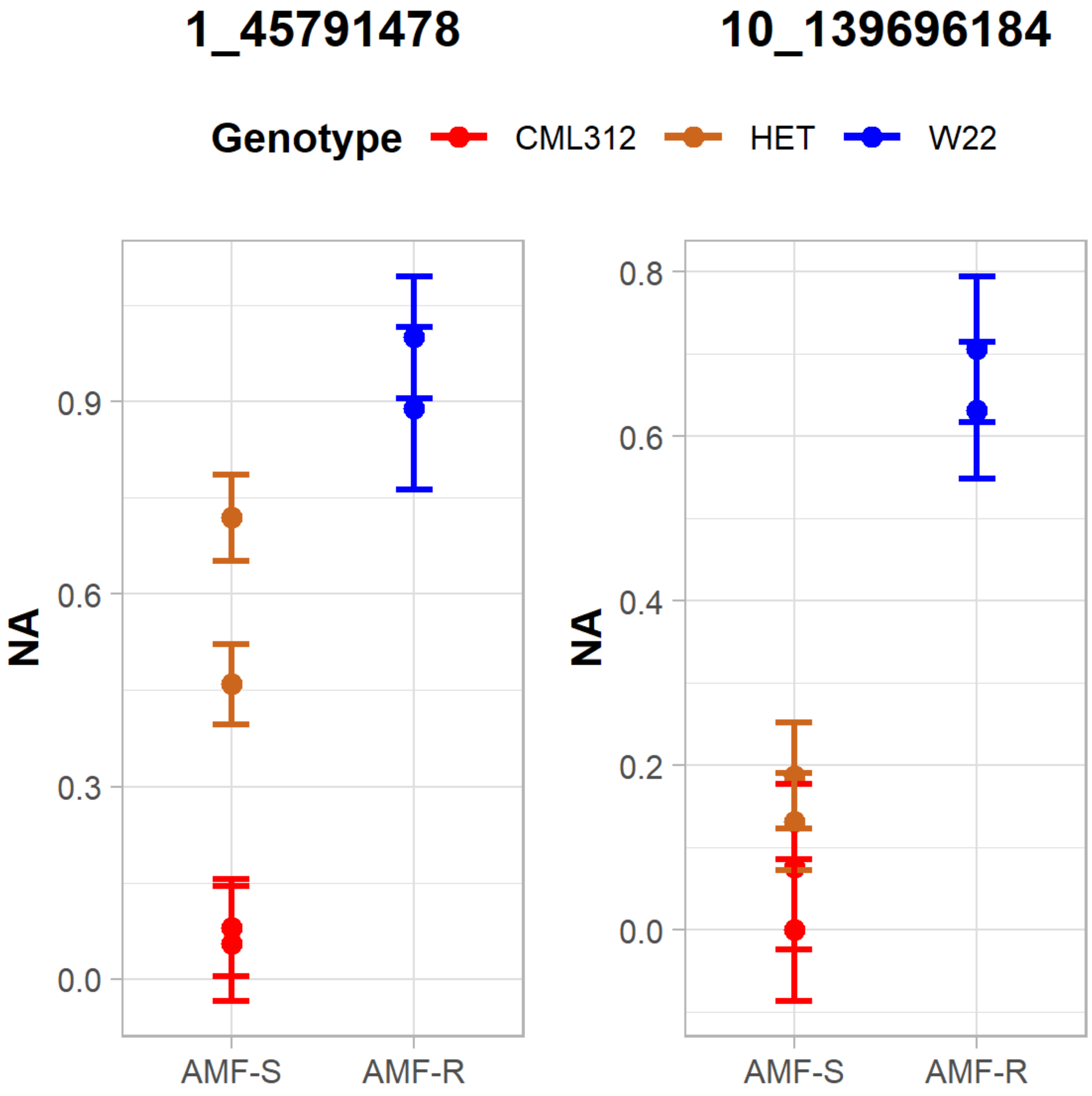
1_45791478
10_139696184
Genotype
CML312
HET
W22
NA
0.0
0.3
0.6
0.9
AMF-S
AMF-R
NA
0.0
0.2
0.4
0.6
0.8
AMF-S
AMF-R

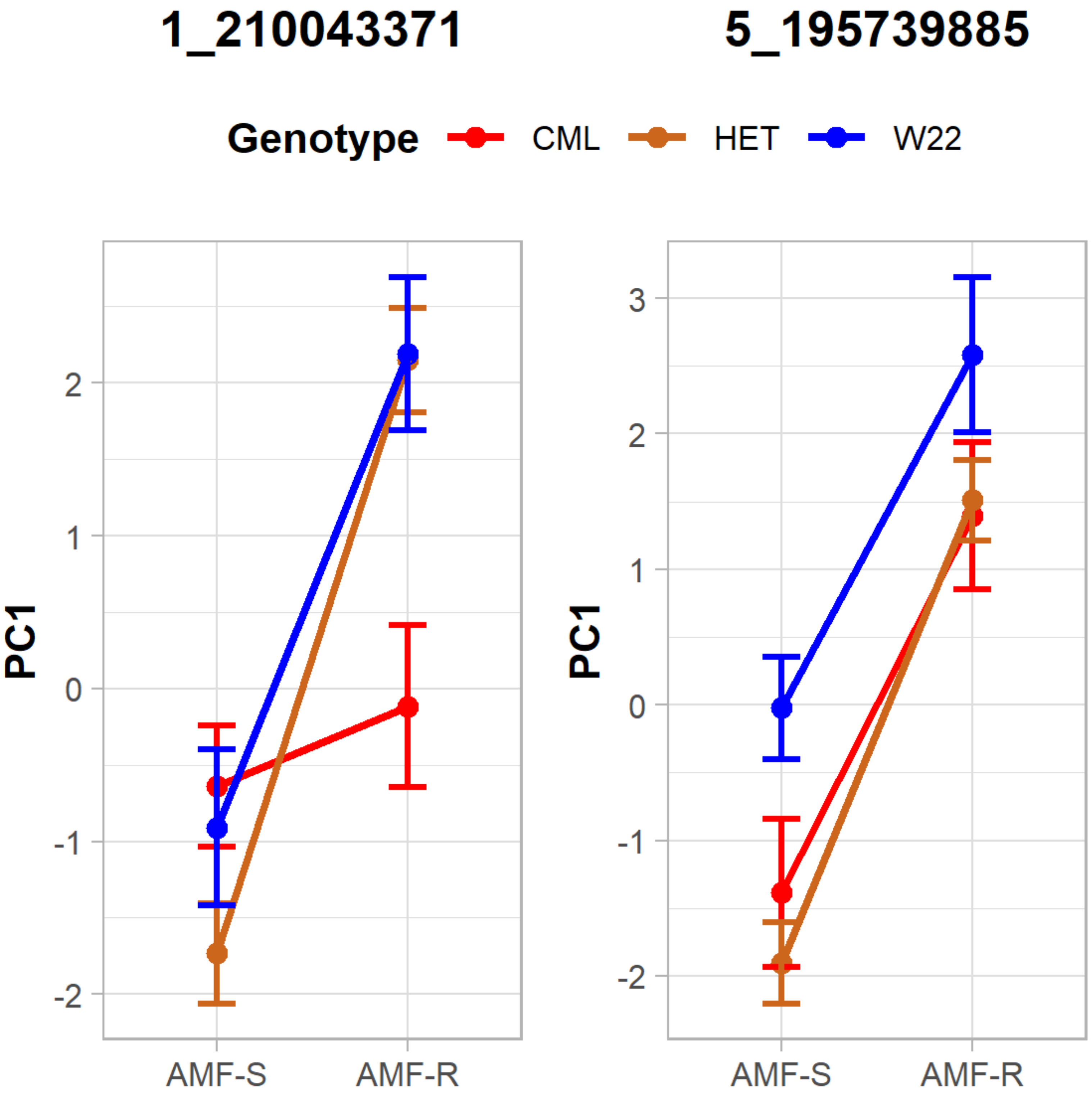
1_210043371
5_195739885
Genotype
CML
HET
W22
PC1
2
1
0
-1
-2
AMF-S
AMF-R
PC1
3
2
1
0
-1
-2
AMF-S
AMF-R

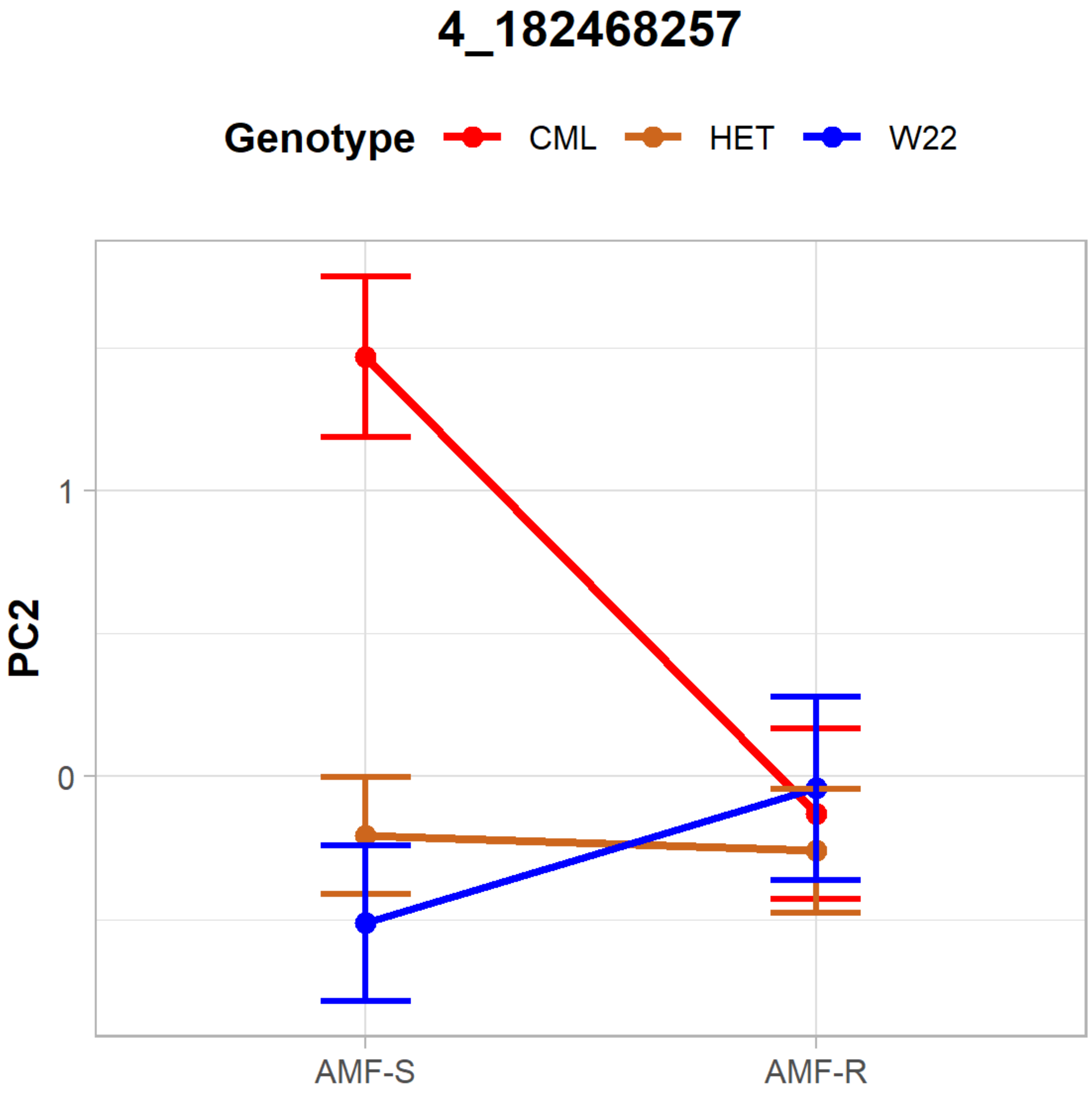
4_182468257
Genotype
CML
HET
W22
PC2
1
0
AMF-S
AMF-R

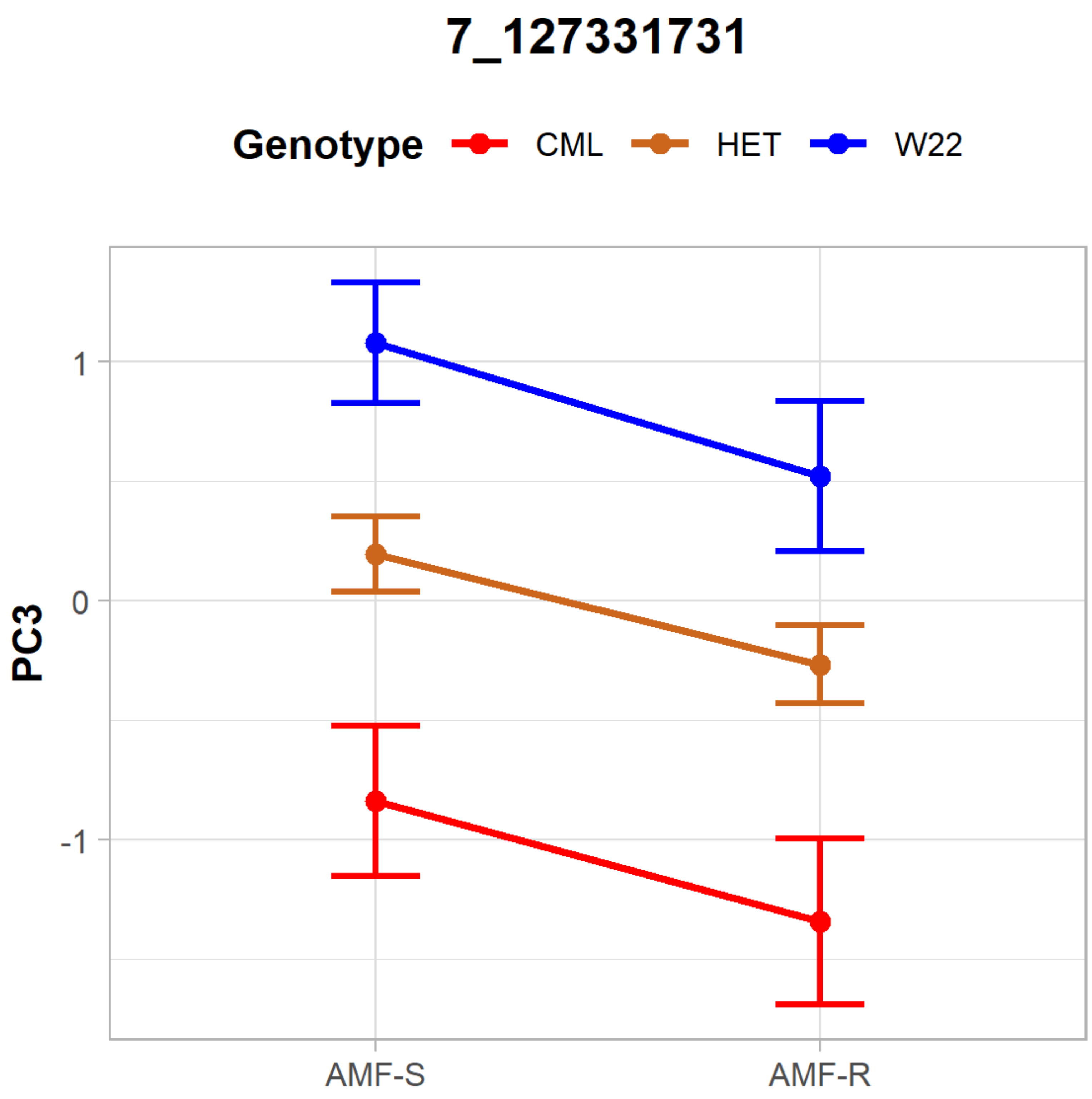

7_127331731
Genotype
CML
HET
W22
PC3
1
0
-1
AMF-S
AMF-R

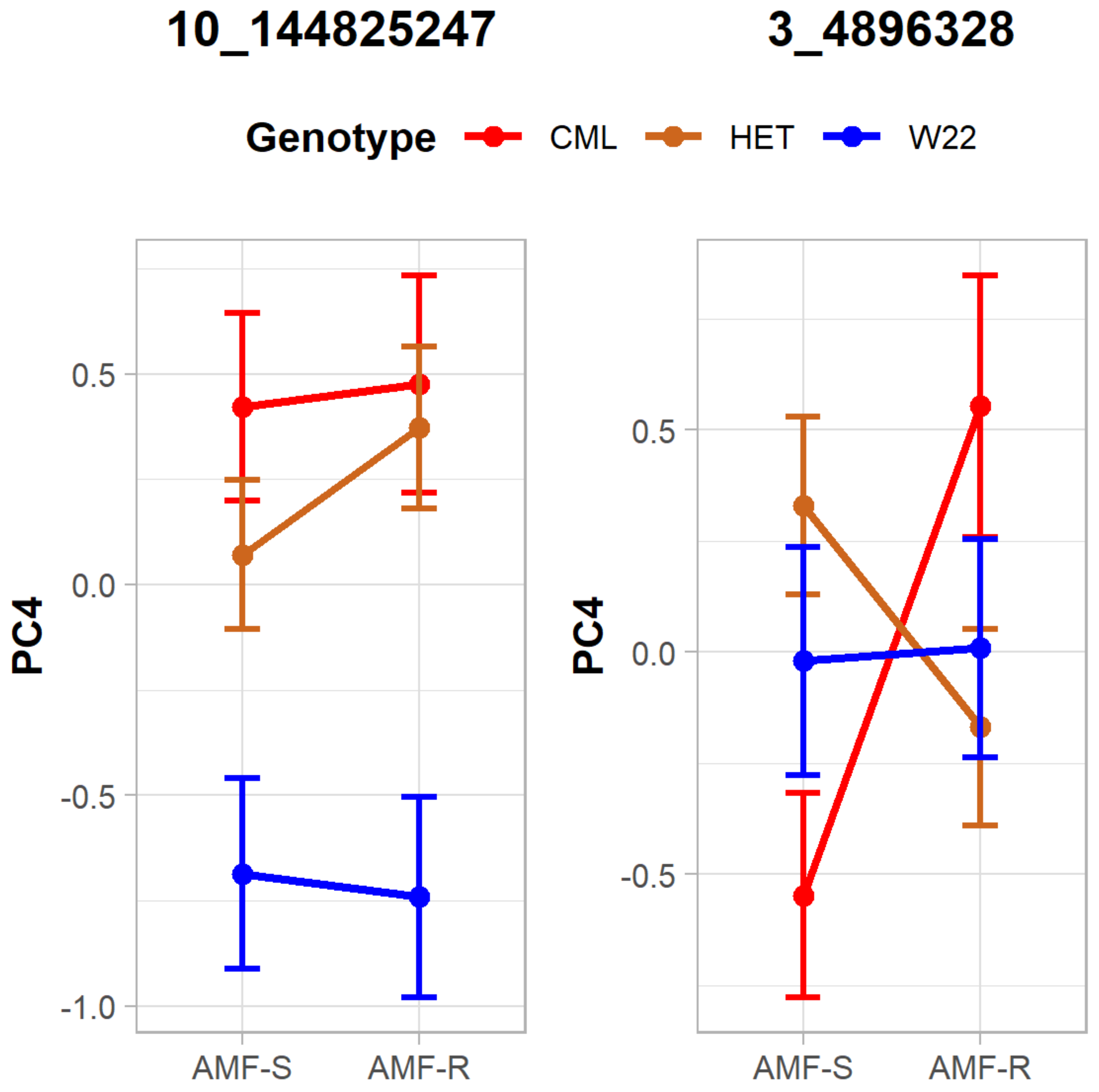

10_144825247
3_4896328
Genotype
CML
HET
W22
PC4
0.5
0.0
-0.5
-1.0
AMF-S
AMF-R
PC4
0.5
0.0
-0.5
AMF-S
AMF-R

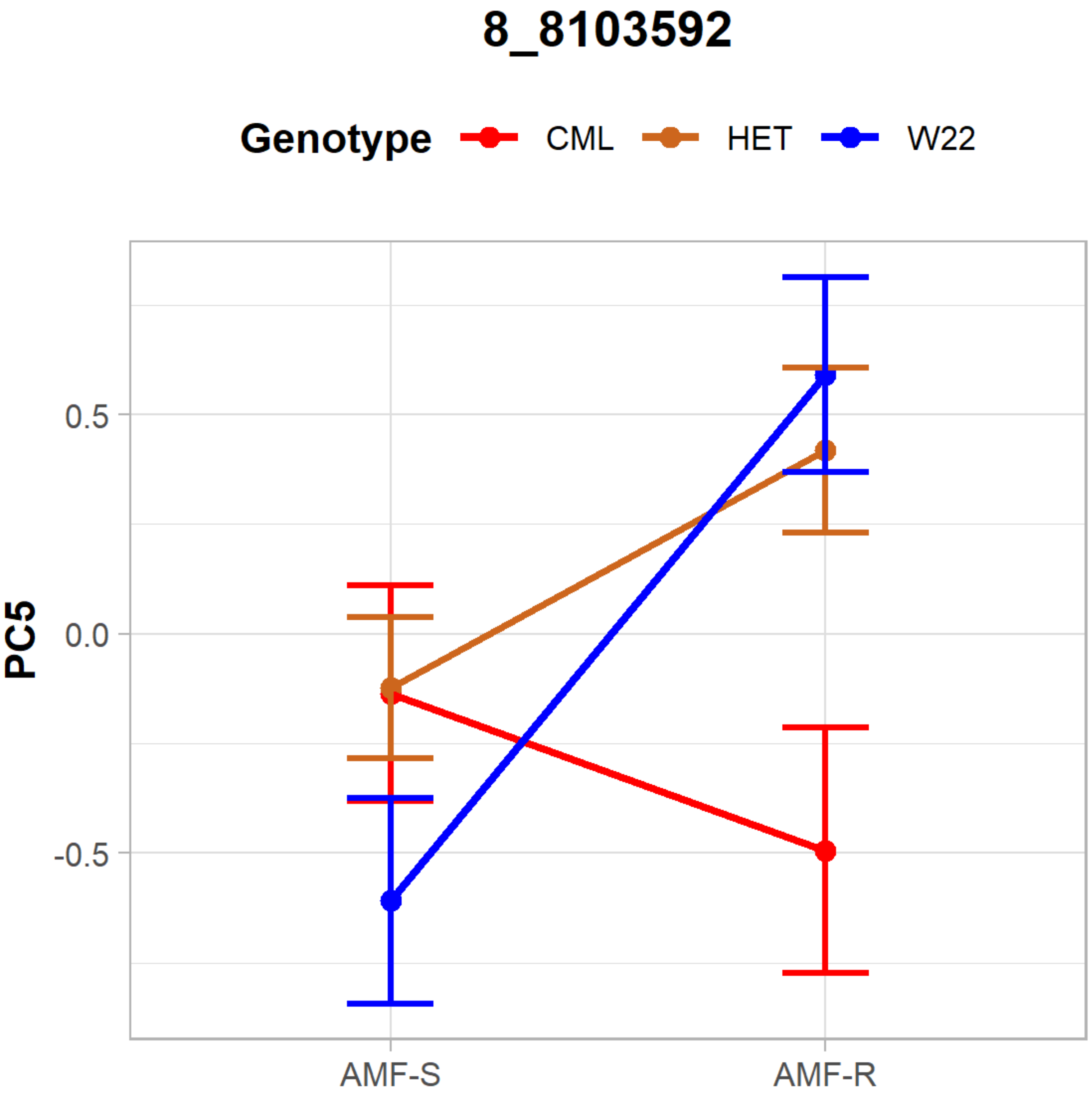
8_8103592
Genotype
CML
HET
W22
PC5
0.5
0.0
-0.5
AMF-S
AMF-R
