# Supplementary material for: The genetic architecture of host response reveals the importance of arbuscular mycorrhizae to maize cultivation
Source: eLife. 2020 Nov 19;9:e61701. doi: 10.7554/eLife.61701 (PMC7676867; doi:10.7554/eLife.61701)
Supplement: Supplementary file 1. [file elife-61701-supp1.docx]

**Supplementary Tables**

**Table S1: Description of phenotypic traits measured in this study**

| **Phenotypic traits** | **Description** | **Unit** |
| --- | --- | --- |
| DTA | Days to anthesis | Day |
| DTS | Days to silking | Day |
| ASI | Anthesis-silking interval | Day |
| PH | Plant height | cm |
| TBN | Tassel branch number | Count |
| EW | Ear weight | g |
| EL | Ear length | cm |
| ED | Ear diameter | cm |
| KRN | Number of kernel rows | Count |
| KPR | Number of kernels per row | Count |
| CD | Cob diameter | cm |
| 50GW | Weight of 50 kernels | g |
| TKW | Total kernel weight | g |
| TKN | Total kernel number | Count |

**Table S2. Broad sense heritability (H^2^) and percentage of phenotypic variance explained by *AMF* additive term (AMF), interaction between *AMF* and the QTLs (QTLxAMF) and QTL additive term (QTL) in the multi-qtl model for each trait .**

| Trait | H2 | AMF | QTLxAMF | QTL |
| --- | --- | --- | --- | --- |
| STD | 41.6 |  |  |  |
| DTA | 81.2 | 0.1 | 6.9 | 6.9 |
| DTS | 61.6 | .2 | 9.4 | 6 |
| ASI | 50.1 | 17.5 | 8.5 | 3.4 |
| PH | 68.2 | 39 | 6.6 | 4 |
| TBN | 75.2 | 4.1 | 6.5 | 15.9 |
| EW | 54.6 | 31.2 | 7.2 | 1.7 |
| EL | 63.1 | 16.3 | 10 | 3.6 |
| ED | 56.8 | 25 | 6 | 5.4 |
| CD | 64.1 |  |  |  |
| KRN | 62.8 |  |  | 11.1 |
| KPR | 55.4 | 43.7 | 6 | 3.5 |
| KC | 70.6 |  |  | 40.5 |
| GC | 79.4 |  |  |  |
| FKW | 59.4 | 10.2 | 6.2 | 4.7 |
| TKW | 57.7 | 25.2 | 10.5 | 0.7 |
| TKN | 52.4 | 30.1 | 6.3 | 2 |
